# Biological and mutational analyses of CXCR4–antagonist interactions and design of new antagonistic analogs

**DOI:** 10.1042/BSR20230981

**Published:** 2023-12-22

**Authors:** Qian Meng, Ruohan Zhu, Yujia Mao, Siyu Zhu, Yi Wu, Lina S.M. Huang, Aaron Ciechanover, Jing An, Yan Xu, Ziwei Huang

**Affiliations:** 1 School of Life Sciences, Tsinghua University, Beijing 100084, China; 2 Division of Infectious Diseases and Global Public Heath, Department of Medicine, School of Medicine, University of California at San Diego, 9500 Gilman Drive, La Jolla, CA 92093, U.S.A.; 3 The Rapport Faculty of Medicine, Technion-Israel Institute of Technology, Haifa 3109601, Israel; 4 Ciechanover Institute of Precision and Regenerative Medicine, School of Medicine, Chinese University of Hong Kong, Shenzhen 518172, China

**Keywords:** chemokine receptor CXCR4, drug design, ligand-receptor interactions, molecular modeling, peptide antagonists, small molecule antagonists

## Abstract

The chemokine receptor CXCR4 has become an attractive therapeutic target for HIV-1 infection, hematopoietic stem cell mobilization, and cancer metastasis. A wide variety of synthetic antagonists of CXCR4 have been developed and studied for a growing list of clinical applications. To compare the biological effects of different antagonists on CXCR4 functions and their common and/or distinctive molecular interactions with the receptor, we conducted head-to-head comparative cell-based biological and mutational analyses of the interactions with CXCR4 of eleven reported antagonists, including HC4319, DV3, DV1, DV1 dimer, V1, vMIP-II, CVX15, LY2510924, IT1t, AMD3100, and AMD11070 that were representative of different structural classes of D-peptides, L-peptide, natural chemokine, cyclic peptides, and small molecules. The results were rationalized by molecular modeling of CXCR4–antagonist interactions from which the common as well as different receptor binding sites of these antagonists were derived, revealing a number of important residues such as W94, D97, H113, D171, D262, and E288, mostly of negative charge. To further examine this finding, we designed and synthesized new antagonistic analogs by adding positively charged residues Arg to a D-peptide template to enhance the postulated charge–charge interactions. The newly designed analogs displayed significantly increased binding to CXCR4, which supports the notion that negatively charged residues of CXCR4 can engage in interactions with moieties of positive charge of the antagonistic ligands. The results from these mutational, modeling and new analog design studies shed new insight into the molecular mechanisms of different types of antagonists in recognizing CXCR4 and guide the development of new therapeutic agents.

## Introduction

The superfamily of G protein-coupled receptors (GPCRs) consists of >800 members, which can be divided into five major groups: glutamate, rhodopsin, adhesion, frizzled/taste2, and secretin [1]. Currently, GPCRs account for a large class of drug targets for many diseases [2]. The CXC chemokine receptor type 4 (CXCR4) is a rhodopsin-like GPCR with seven transmembrane (TM) helices, which can trigger a series of signal pathways upon binding its agonist ligands [3], leading to many physiological functions including hematopoiesis [4], embryogenesis [5], and vascularization [6] and being implicated in human diseases such as cancer growth and metastasis [7–9] and HIV-1 infection [10].

CXCR4 and C-C chemokine receptor type 5 (CCR5) are co-receptors for HIV-1, which recruits CD4 as a primary receptor and anchors to human cells [11]. M-tropic strains of HIV-1, which use CCR5 as a co-receptor, participate in the early stage of HIV-1 infection whereas T-tropic strains of HIV-1 using another co-receptor CXCR4 are involved in the late stage of viral infection [12]. Initial steps of HIV-1 infection involve the interactions of HIV-1 envelope protein gp120 with CD4 and CXCR4 and/or CCR5 on host cells, rendering such interactions promising targets for blocking HIV-1 entry. A HIV-1 entry inhibitor, maraviroc (also known as Selzentry or MVC) that is clinically approved by the U.S. Food and Drug Administration (FDA) is a CCR5-targeting small molecule shown to reduce HIV-1 infection in patients. The limitation is that this drug blocks HIV-1 strains that use only CCR5 as a co-receptor but not those strains which use CXCR4 or both. A fusion inhibitor enfuvirtide (T-20) clinically approved by FDA prevents the fusion of HIV-1 with the host cell membrane by targeting the virus’s envelope protein gp41 and is not dependent on co-receptor usage [13]. So far, a drug directly targeting the CXCR4 co-receptor for the treatment of HIV-1 infection is not yet available clinically.

SDF-1α (also known as CXCL12) is the cognate agonist chemokine that binds CXCR4 and mediates its functions during the migration of hematopoietic cells and embryogenic events [5]. SDF-1α has been shown to prevent the entry of HIV-1 via CXCR4 [14,15], which demonstrates the therapeutic potential of targeting the SDF-1α–CXCR4 axis for HIV-1 inhibition. Mutational analysis studies of SDF-1α–CXCR4 interaction and signal transduction have been reported, which provide mechanistic insights into CXCR4–agonist interaction and signal transmission [16,17]. In addition to the cognate agonist (i.e. SDF-1α) known for CXCR4, there are natural antagonists such as vMIP-II produced by human herpesvirus-8, many different types of synthetic antagonists [18], as well as non-cognate agonists including macrophage migration inhibitory factor (MIF) [19,20] and extracellular ubiquitin reported for this receptor. These antagonistic molecules, capable of inhibiting the functions of SDF-1α and CXCR4 in various diseases and clinical applications, have provided diverse templates for the development of CXCR4-targeted therapeutics and thus been the subjects of extensive structure–function studies of their interactions with CXCR4.

Structure-function analysis studies have led to the notion that the 7 TM helices in CXCR4 and other chemokine receptors can form two pockets, a major pocket consisting of TM helices III, IV, V, VI, and VII and a minor pocket consisting of TM helices I, II, III, and VII [21]. The crystal structures of CXCR4 in complex with three different antagonists, IT1t, CVX15, and vMIP-II have revealed the binding of IT1t (a small molecule) to the minor pocket of CXCR4, CVX15 (a synthetic peptide of the size between IT1t and vMIP-II) to the major pocket, and vMIP-II (a natural chemokine of the largest size among these three) to both the major and minor pockets [22,23]. While the co-crystal structures provide important insights into the receptor recognition mechanisms of these three antagonists, such structural information is not yet available for many other CXCR4 antagonists reported in the literatures, thus leaving the receptor binding mechanisms of these antagonists subject to further structure–function studies.

To understand the receptor–ligand recognition mechanism involving CXCR4 and its antagonists, here in this study we conducted a series of comparative analyses of the biological activities and CXCR4 binding sites of eleven known CXCR4 antagonists, including the three mentioned above, using cell-based bioassays, CXCR4 site-directed mutagenesis and computer-aided molecular modeling. These antagonists studied here were chosen from different representative structural types including both D-peptides (i.e., HC4319, DV1, DV1 dimer, and DV3) and a representative counterpart L-peptide (i.e., V1) derived from the N-terminus of vMIP-II [24–27]; the full-length natural ligand vMIP-II with an IC_50_ of 5.8 nM [28]; two cyclic peptides: CVX15 with a competitive binding IC_50_ of 6 nM and LY2510924 with subnanomolar binding affinity [23,29,30]; and three small molecules: IT1t of competitive binding IC_50_ of 8 nM and calcium mobilization inhibitory IC_50_ of 1.1 nM, AMD3100 of submicromolar potency, and AMD11070 which is a nanomolar potent derivative of AMD3100 [31–33]. Among these, AMD3100 is the only CXCR4 antagonist approved by the FDA so far for clinical use as a mobilizer of hematopoietic stem cells in the treatment of non-Hodgkin’s lymphoma and multiple myeloma [34–36]. AMD11070 is a non-cyclam analog of AMD3100 for which clinical trials for anti-HIV treatment were conducted but discontinued due to side-effects in the livers [37]. More recently, AMD11070 was granted the orphan drug status and entered a Phase III clinical trial for the treatment of warts, hypogammaglobulinemia, infections, and myelokathexis (WHIM) syndrome. LY2510924 was also in clinical trials for treating multiple diseases such as solid tumors, acute myeloid leukemia (AML), extensive-disease small cell lung cancer (ED-SCLC), and advanced cancer [29,30,38–42]. Other compounds used in this study were in the preclinical stage, including linear L- and D-peptides derived from vMIP-II, shown by our laboratories to bind CXCR4 and to have potent activity in animal models of AML and hematopoietic stem cells mobilization [43,44]. Additionally, small molecule IT1t effectively blocks X4-tropic HIV infection via CXCR4 with an IC_50_ of 7 nM and reduces inflammation in mice [31,45]. Futhermore, cyclic peptide CVX15, an analog of T140, binds the major subpocket of CXCR4, as revealed by co-crystal structure with CXCR4 [23]. We carried out comparative studies for this panel of eleven CXCR4 antagonists with vastly different structures and molecular weights and reported by different laboratories or companies. Their receptor antagonizing and inhibitory activities were assessed in parallel in cell-based CXCR4 competitive binding, calcium influx, cell migration, and CXCR4 internalization assays. Next, their receptor binding sites on CXCR4 TM domains were determined using a panel of CXCR4 site-directed mutants. The obtained biological and mutational data was analyzed and rationalized by computer-aided molecular modeling to gain insights into the structure–function relationship and mechanism of binding of these antagonists with the CXCR4 receptor. The results reported here provide experimental comparison of a large and diverse panel of CXCR4 antagonists in their cellular bioactivities and receptor binding modes which can be used to design new analogs with stronger receptor binding. The key CXCR4 residues important for the binding of diverse ligands were found to often include negatively charged residues such as D97, D171, D262 and E288. As such, we designed new analogs of a representative D-peptide antagonist DV3 by adding positively charged Arg residues to DV3 to enhance its interactions with the above-mentioned negatively charged residues on CXCR4. This resulted in stronger receptor binding of the new analogs which supported the design notion.

## Results

### Selection of CXCR4 antagonists for comparative studies

For a systematic analysis of inhibitory activities and receptor binding modes, we selected a panel of 11 CXCR4 antagonists based on the consideration of structural diversity and representativeness. As shown in Table 1, this included a full-length, natural chemokine vMIP-II of the largest size and highest molecular weight, relatively smaller peptide V1 derived from the N-terminus of vMIP-II, unnatural D-amino acid containing D-peptides DV1 and DV3, dimeric D-peptides DV1 dimer and HC4319, cyclic peptides CVX15 and LY2510924, and non-peptidic small molecules IT1t, AMD3100, and AMD11070 of the lowest molecular weights.

**Table 1:** Sequences and structures of eleven antagonists

CXCR4 antagonists	Sequence*/structure	IC_50_ (nM)
HC4319	Leu-Gly-Ala-Ser-Trp-His-Arg-Pro-Asp-Lys-Cys-Cys-Leu-Gly-Tyr-Gln-Lys-Arg-Pro-Leu-Pro-Lys	46.0 ± 12.6
	Leu-Gly-Ala-Ser-Trp-His-Arg-Pro-Asp-Lys	
DV1	Leu-Gly-Ala-Ser-Trp-His-Arg-Pro-Asp-Lys-Cys-Cys-Leu-Gly-Tyr-Gln-Lys-Arg-Pro-Leu-Pro	364.7 ± 51.7
DV3	Leu-Gly-Ala-Ser-Trp-His-Arg-Pro-Asp-Lys	2596.6 ± 422.4
DV1 dimer	Leu-Gly-Ala-Ser-Trp-His-Arg-Pro-Asp-Lys-Cys-Cys-Leu-Gly-Tyr-Gln-Lys-Arg-Pro-Leu-Pro	60.5 ± 12.8
	Leu-Gly-Ala-Ser-Trp-His-Arg-Pro-Asp-Lys-Cys-Cys-Leu-Gly-Tyr-Gln-Lys-Arg-Pro-Leu-Pro	
V1	Leu-Gly-Ala-Ser-Trp-His-Arg-Pro-Asp-Lys-Cys-Cys-Leu-Gly-Tyr-Gln-Lys-Arg-Pro-Leu-Pro	2632.1 ± 891.0
vMIP-II	LGASWHRPDKCCLGYQKRPLPQVLLSSWYPTSQLCSKPGVIFLTKRGRQVCADKSKDWVKKLMQQLPVTAR	10^†^
CVX15	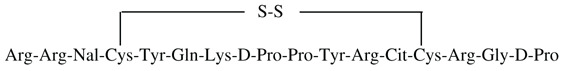	7.8 ± 2.2
LY2510924	Cyclo[Phe-Tyr-Lys(iPr)-D-Arg-2-Nal-Gly-D-Glu]-Lys(iPr)-NH2	135.4 ± 63.9
IT1t	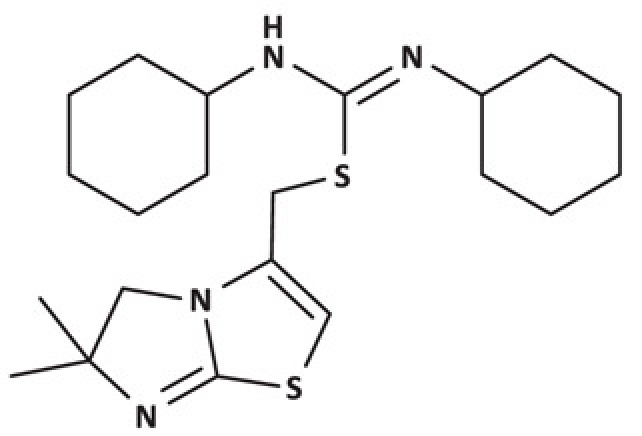	29.65 ± 2.8
AMD3100	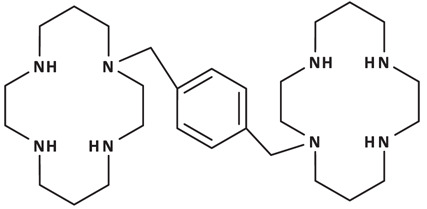	319.6 ± 37.3
AMD11070	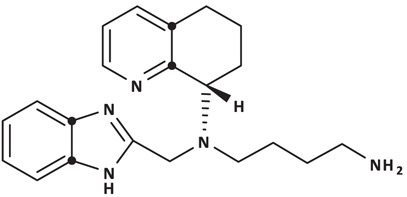	15.6 ± 7.6
HM60303	Lys-Arg-Leu-Gly-Ala-Ser-Trp-His-Arg-Pro-Asp-Lys	>50,000
HM60323	Leu-Gly-Ala-Ser-Trp-His-Arg-Pro-Asp-Lys-Arg	706.6 ± 143.2
HM12	Leu-Gly-Ala-Ser-Trp-His-Arg-Pro-Asp-Lys-Arg-Arg	544.0 ± 75.0
HM13	Leu-Gly-Ala-Ser-Trp-His-Arg-Pro-Asp-Lys-Nal-Arg-Arg	377.4 ± 7.7
HM70116	Leu-Gly-Ala-Ser-Trp-His-Arg-Pro-Nal-Arg-Arg	193.9 ± 37.9

* Sequences of the D-peptides HC4319, DV1, DV1 dimer, DV3, HM60303, HM60323, HM12, HM13, and HM70116 are composed of D-amino acids. The sequence of vMIP-II is shown by one letter code.

† The IC_50_ value of vMIP-II was from the previously published results [42]. The IC_50_ values of CXCR4 antagonists were averages of at least three independent experiments shown as mean ± standard error of mean (S.E.M.).

### Competitive CXCR4 binding activity

**Figure 1:**
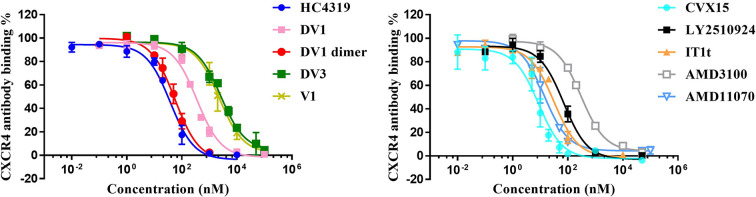
Competitive CXCR4 binding curves of eleven CXCR4 antagonists The CXCR4 transfected CHO cells were incubated with different concentrations of antagonists and 250 ng/ml 12G5 antibody. The CXCR4-binding 12G5 antibody was recognized by FITC-conjugated anti-IgG second antibody. The cells treated with only second antibody were used as a negative control (0%) while cells treated with both 12G5 antibody and second antibody were used as a positive control (100%). Cells treated with CXCR4 antagonists were calculated in percentage relative to the positive and negative controls. The data were averages of at least three independent experiments and shown as mean ± standard error of the mean (S.E.M.).

The CXCR4-specific antibody, 12G5 was used as a probe for the competitive CXCR4 binding assay to evaluate the binding activity of CXCR4 antagonists. As shown in Table 1 and Figure 1, a range of CXCR4 binding activities were displayed by these antagonistic molecules shown by the IC_50_ values in inhibiting 12G5 binding: D-peptides HC4319 (46.0 ± 12.6 nM), DV1 (364.7 ± 51.7 nM), DV3 (2596.6 ± 422.4 nM), and DV1 dimer (60.5 ± 12.8 nM), L-peptide V1 (2632.1 ± 891.0 nM), vMIP-II (10 nM) [46], cyclic peptides CVX15 (7.8 ± 2.2 nM), and LY2510924 (135.4 ± 63.9 nM), and small molecule inhibitors IT1t (29.65 ± 2.8 nM), AMD3100 (319.6 ± 37.3 nM), and AMD11070 (15.6 ± 7.6 nM).

### Inhibition of CXCR4-mediated cell migration

**Figure 2:**
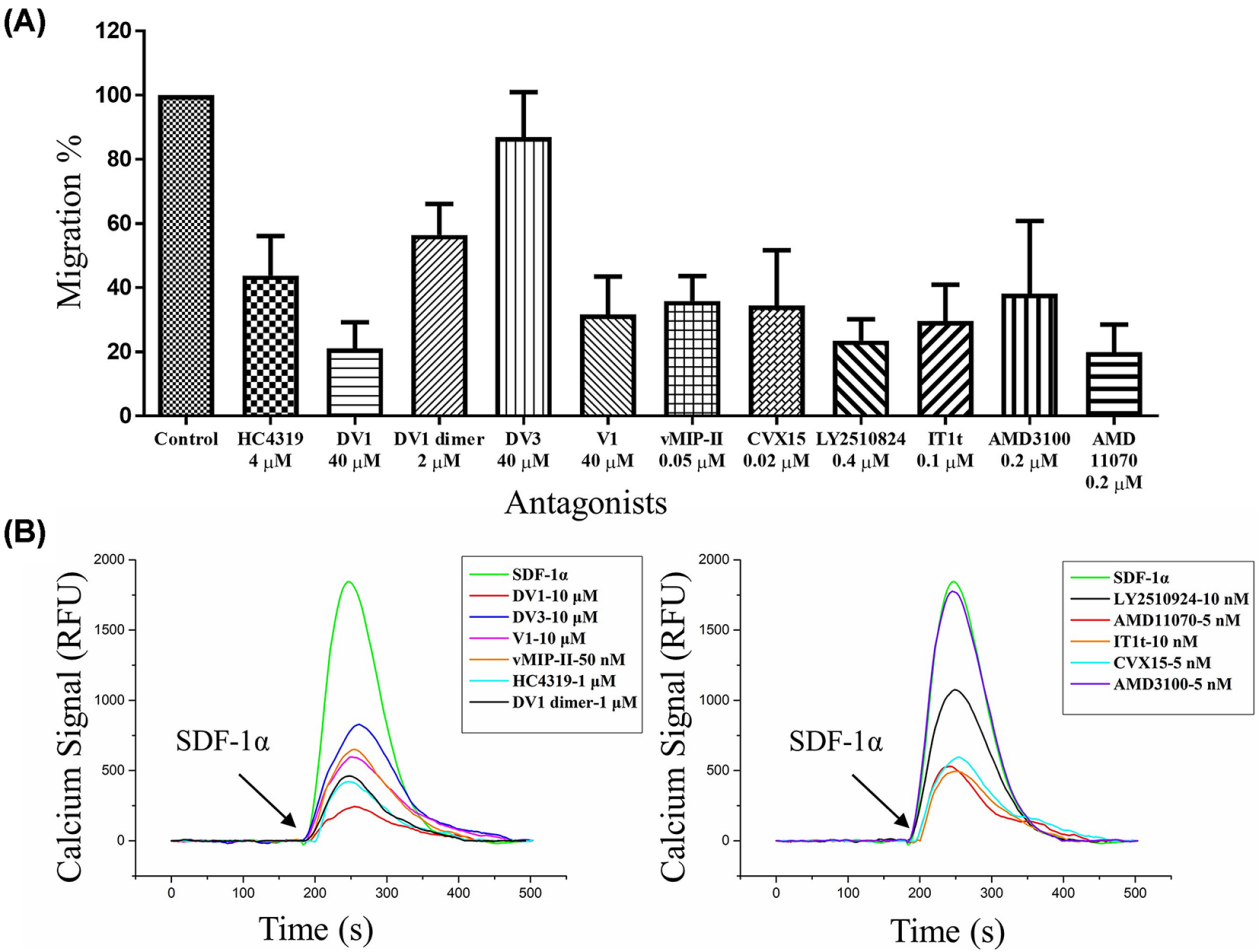
The inhibitory effect of the antagonists on SDF-1α-induced cell chemotaxis and calcium mobilization in SupT1 cells The antagonists and concentrations used were labeled in each lane. (**A**) The inhibition of cell migration by the noted antagonists. SupT1 cells pre-treated with or without CXCR4 antagonists were seeded in the upper well and cell migrated by 2 nM SDF-1α. Migrated cells without SDF-1α were used as a negative (0%) and migrated cell induced by SDF-1α as a positive control (100%). The data were displayed as mean ± standard deviation (S.D.) (**B**) The inhibition of CXCR4 antagonists on SDF-1α-CXCR4 induced intracellular calcium mobilization. SupT1 cells were treated with or without CXCR4 antagonists and activated by 50 nM SDF-1α. To directly compare the inhibitory effect of the antagonists on calcium mobilization, the vehicle background and baseline were removed using GraphPad and Origin. The data were averages of at least three independent experiments.

Upon the binding of SDF-1α, CXCR4 activates multiple signal pathways and mediates cell movement [47], which can be inhibited by the antagonists. As shown in Figure 2A, the D-peptides inhibited 56% of cell migration at 4 μM for HC4319, 43% at 2 μM for DV1 dimer, and 13% at 40 μM for DV3. DV1 inhibited 78% of cell migration at 40 μM while its L-peptide counterpart V1 inhibited 68% of cell migration at the same concentration. The herpes virus encoded chemokine vMIP-II could inhibit 64% of cell migration at 50 nM. The cyclic peptide CVX15 exhibited potent inhibition of 65% of cell migration at 20 nM while another cyclic peptide LY2510924 could inhibit 76% of cell migration at 400 nM. The small molecule IT1t could inhibit 70% of cell migration at 100 nM while AMD3100 and AMD11070 could inhibit 61% and 80% of cell migration at 200 nM, respectively.

### Inhibition of SDF-1α-induced calcium mobilization

Upon binding CXCR4, SDF-1α induces transient calcium concentration increase [48]. The inhibitory effects of the antagonists on calcium mobilization are shown in Figure 2B. For most antagonists, a single concentration was used, except for AMD3100 on which multiple concentrations were examined to derive AMD3100’s inhibitory curve and IC_50_ in inhibiting SDF-1α-CXCR4 induced intracellular calcium mobilization (Supplementary Figure S1). The choice of the single concentration for each antagonist was made based on the CXCR4 competitive binding potency (the IC_50_ values described above) and varied between different groups of compounds with structural similarity. For examples, we chose the same concentration for HC4319 and DV1 dimer (both are D-bivalent peptides), DV1 and DV3 (both are D-peptides derived from vMIP-II N-terminus) and V1 (L-peptide with identical sequence to DV1). Under the same consideration, the same single concentration of AMD3100 and its structural analog AMD11070 was tested to compare their different potencies at the same concentration. As detailed below, this allowed for a quick and less costly initial assessment of different antagonists within the same structural groups under the same tested concentrations.

The D-peptides HC4319 and DV1 dimer could inhibit 77% and 75% of calcium mobilization at 1 μM, respectively. DV1 inhibited 86% of the calcium signal at 10 μM and was more potent than its L-peptide counterpart V1. DV3 inhibited about 55% of the calcium signal at 10 μM. Not surprisingly, vMIP-II could effectively inhibit 64% of the calcium signal even at a relatively low concentration of 50 nM. AMD3100 at 5 nM showed very weak inhibition of calcium mobilization compared with AMD11070 which had 71% inhibition. Previous studies by others showed that AMD3100 inhibited the calcium mobilization with an IC_50_ of 572 nM [49] whereas AMD11070 was more potent with an IC_50_ of 9 nM [50]. This may explain the observation here in our study where AMD3100 had little effect at 5 nM, which was much lower than the effective concentration of 572 nM mentioned in the literature and in our study while AMD11070 showed strong effect at 5 nM that was close to its IC_50_ value reported by others. Another small molecule IT1t and the two cyclic peptides CVX15 and LY2510924 all displayed significant inhibition of the calcium mobilization.

### Inhibition of CXCR4 internalization

**Figure 3:**
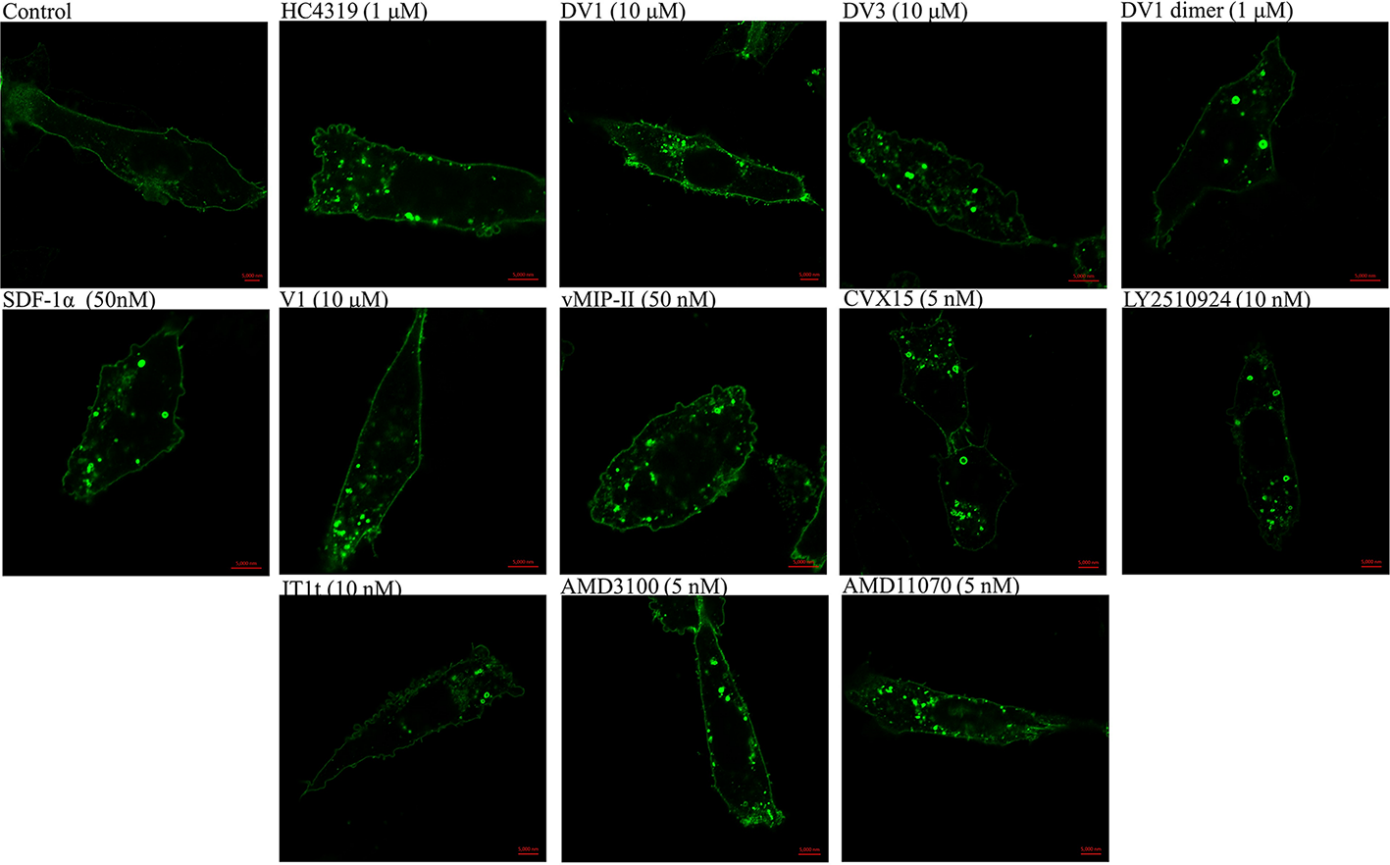
The inhibitory effect of the antagonists on CXCR4 internalization CHO cells expressed CXCR4-EGFP conjugates were seeded in 96-well plate with glass bottom. Untreated cells were used as a control. Cells were pre-treated with or without CXCR4 antagonists and internalized with 50 nM SDF-1α (scale bar: 5 μm). The concentrations of the antagonists used were the same as those for the calcium mobilization assay shown above. The data shown were representative of three independent experiments.

When activated by SDF-1α, CXCR4 undergoes clathrin-dependent endocytosis [51], which can be detected by the internalization of CXCR4-EGFP conjugate and the formation of clathrin-coated vesicles. As shown in Figure 3, at the same concentrations used in the calcium mobilization assay described above, the inhibitory effect could be evaluated by the the numbers and diameters of the formed vesicles, among tested antagonits, LY2510924 and DV1 dimer which showed lessened effect on blocking CXCR4 endocytosis compared with their inhibitory effect on the calcium mobilization.

### Determination of antagonist’s binding sites on CXCR4 by site-directed mutagenesis

**Figure 4:**
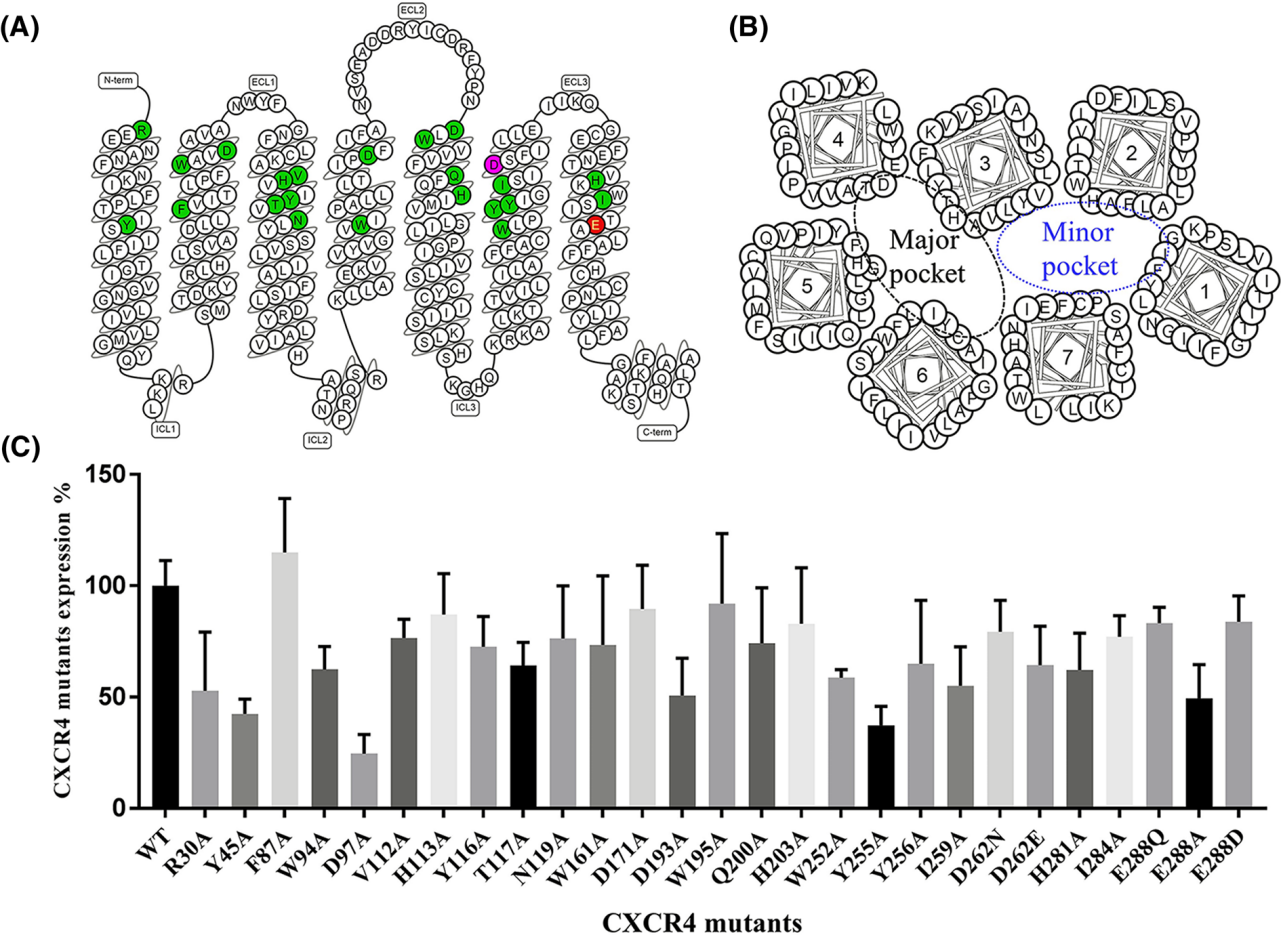
Mutated residues of CXCR4 and cell surface expression of CXCR4 wild-type and mutants (**A**) The schematic representation of serpentine CXCR4 transmembrane helices and loops is shown, highlighting residues colored in green that were mutated to alanine, a residue in magenta mutated to asparagine and glutamate, and a residue in red mutated to glutamine, alanine, and aspartic acid. (**B**) The major and minor pockets of CXCR4 for antagonist binding are shown. (**C**) The cell surface expression levels of wild-type and mutant CXCR4 are shown. CHO cells stably expressing CXCR4 wild-type and mutants were stained with a CXCR4 specific antibody 12G5 for fluorescence intensity analysis. Bars represent the relative fluorescence intensity ratio between CXCR4 wild-type and mutants. The expression levels are shown as mean ± S.D. from at least three independent experiments.

The interactions of CXCR4 with these eleven antagonists were analyzed by site-directed mutagenesis to determine residues on the 7 TM domains of CXCR4 that are important for antagonist's binding. Twenty-four residues of CXCR4 were selected for point mutation to alanine mostly or in a few cases other amino acids: R30A, Y45A, F87A, W94A, D97A, V112A, H113A, Y116A, T117A, N119A, W161A, D171A, D193A, W195A, Q200A, H203A, W252A, Y255A, Y256A, I259A, D262N, D262E, H281A, I284A, E288Q, E288A, and E288D (Figure 4A). Among these CXCR4 mutated residues, it is known that D171, D262 and E288 are important for AMD3100’s binding to CXCR4 [52–54], that W94, D97 and E288 are important for IT1t’s binding to CXCR4 [23], and that other residues are components of the CXCR4 binding pocket and responsible for multiple antagonists’ binding to CXCR4 [22,23]. These mutated residues are distributed in both the major and minor pockets of CXCR4 (Figure 4B). All of these point mutations were confirmed by sequencing. The wild-type and mutated CXCR4 constructs were stably transfected into CHO cells. CXCR4 expression was assessed by 12G5 antibody staining, followed by recognition using FITC-labeled second antibody, and quantification using a multimode plate reader. We chose 12G5 antibody as a reference for competitive binding as 12G5 recognizes the N-terminus and extracellular loop ECL2 of CXCR4 including residues E2, C28, E179, D181, D182, Y190 and C274 [55]. Our mutant positions didn't include these binding sites and thus the mutations had no influence on the binding affinity of 12G5 antibody to CXCR4. Some of the positions mutated in this study, such as Y45, W94, D97, Y116, W252, H281 and E288, were also studied by others previously [16]. The mutants H113A, D171A, W195A, H203A, E288Q, and E288D showed a similar expression level, over 80%, as wild-type CXCR4; F87A showed a higher expression level compared with wild-type CXCR4; mutants W94A, V112A, Y116A, T117A, N119A, W161A, Q200A, Y256A, D262N, D262E, H281A, and I284A showed relatively lower expression levels compared with wild-type CXCR4 (between 60 and 80%); mutants R30A, Y45A, D97A, D193A, W252A, Y255A, I259A, and E288A showed relatively poor expression levels, below 60%, compared with wild-type CXCR4 (Figure 4C).

The above described 27 CXCR4 mutants and wild type CXCR4 as a comparison were used to perform competitive receptor binding analyses of each of eleven antagonists. As shown in Table 2, varied effects due to the point mutations were observed for these different antagonists. The decrease or loss in receptor binding of an antagonist for a particular point mutation would indicate the important role of the mutated residue for the receptor binding interaction of such an antagonist. The systematic examination of each of eleven antagonists across the whole panel of 27 CXCR4 mutants allowed us to determine the residues (or sites) on CXCR4 involved in the binding of these antagonists. Below, the results and analyses about CXCR4 binding sites are presented for these 11 antagonists divided into four different groups based on molecular types and characters: linear D-peptides, a linear L-peptide and a natural chemokine vMIP-II, cyclic peptides, and non-peptidic small molecules.

**Table 2:** Competitive receptor binding activities of various antagonists for wild-type CXCR4 and its mutants^*^

CXCR4 TM helices	CXCR4 mutations	HC4319	DV1	DV1 dimer	DV3	V1	vMIP-II	CVX15	LY2510924	IT1t	AMD3100	AMD11070
	WT	+++	+++	+++	+++	+++	+++	+++	+++	+++	+++	+++
TM1	R30A	+++	+++	+++	+++	+++	+++	+++	+++	+++	+++	+++
	Y45A	+	++	++	++	++	+++	+++	+	+++	++	++
TM2	F87A	-	-	+	+	+	+	+	-	-	++	+
	W94A	-	-	+	+	+	+	+++	-	+	-	++
	D97A	++	++	++	+	++	++	+++	+	-	++	+
TM3	V112A	+++	+++	+++	+++	+++	+++	+++	++	+++	+++	+++
	H113A	++	++	+	+	+++	+++	+++	+	+	++	+
	Y116A	-	-	+	++	+	++	++	-	+	++	+++
	T117A	+++	+++	++	+++	+++	+++	+++	+	+++	+++	+++
	N119A	-	-	+	++	+	++	+++	+	++	+++	+++
TM4	W161A	+++	++	+++	+++	++	+++	+++	++	+++	+++	++
	D171A	+++	+++	++	++	+++	+++	-	-	+++	+	+
	D193A	++	+++	++	+++	++	++	+++	+++	+++	++	+++
	W195A	++	+	+++	++	++	++	+++	++	++	+++	++
TM5	Q200A	+++	++	+++	+++	+++	+++	+++	-	+++	+++	+++
	H203A	+++	++	++	++	+++	+++	+++	+	+++	+++	+++
TM6	W252A	+++	+++	+++	+++	+++	+++	+++	++	+++	+++	+++
	Y255A	++	+++	+++	+++	+++	+++	+++	++	+++	++	+++
	Y256A	+++	+++	+++	+++	+++	+++	+++	++	+++	+++	+++
	I259A	+++	++	+++	+++	+++	+++	+++	-	+++	+++	+++
	D262N	++	++	+	++	++	+++	+	+++	+++	-	+++
	D262E	++	++	++	++	++	++	++	++	+++	+	+++
TM7	H281A	++	++	++	+++	+	+++	+++	++	+++	+++	++
	I284A	+++	+++	+++	+++	+++	+++	+++	++	+++	+++	+++
	E288Q	++	+	+	++	++	++	+	-	-	-	-
	E288A	+	+	+	+	++	+++	+++	-	+	-	-
	E288D	+	+	++	++	++	+++	+++	-	+	-	-

* The competitive receptor binding activities of various antagonists to different CXCR4 mutants are indicated by +++ (comparable with wild-type CXCR4), ++ (10–29% reduction compared with wild-type CXCR4), + (30–60% reduction compared with wild-type CXCR4) and – (61–100% reduction compared with wild-type CXCR4). The results were averages of at least three independent experiments.

### The CXCR4 binding sites of linear D-peptides HC4319, DV1, DV1 dimer, and DV3

**Figure 5:**
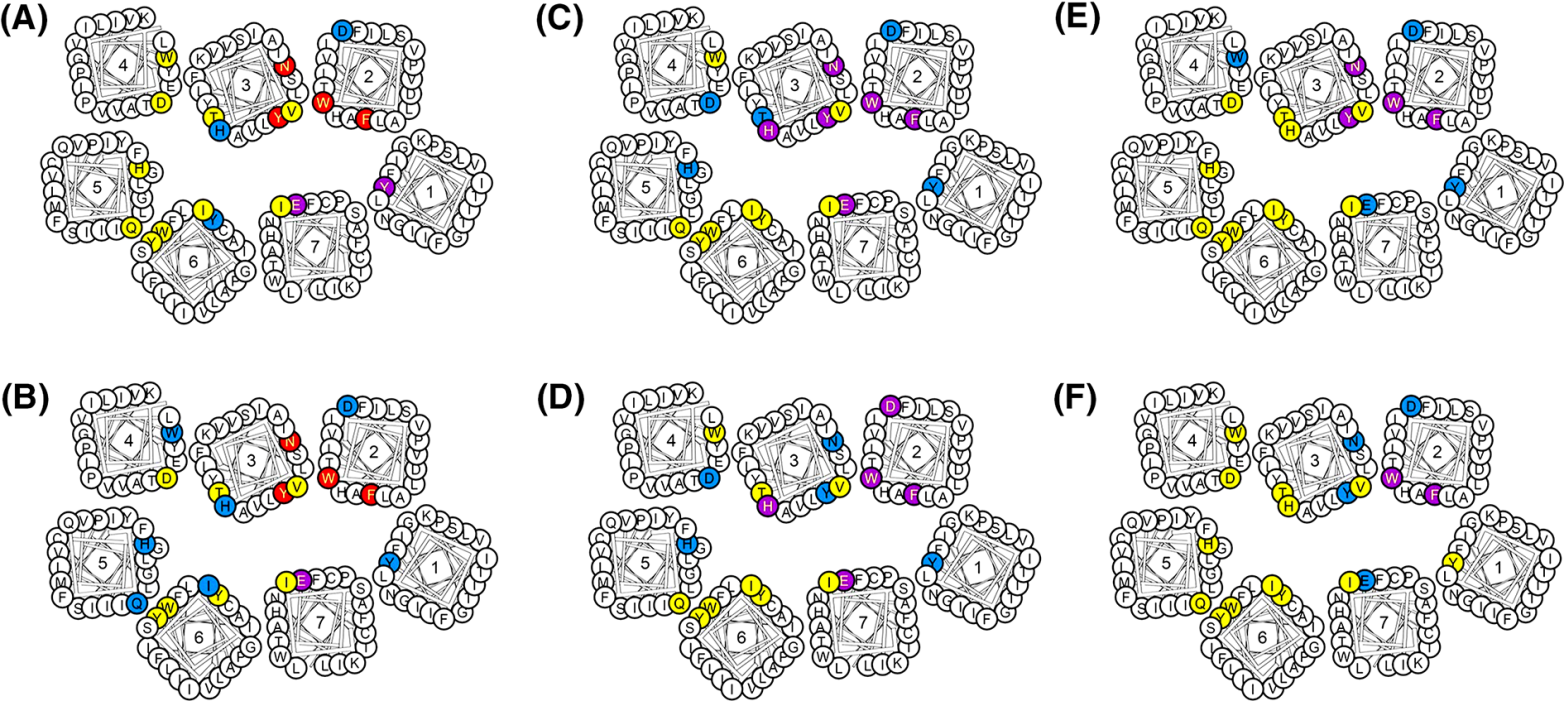
The CXCR4 binding sites of D-peptides, L-peptide V1, and vMIP-II The residues implicated in antagonist binding as determined from mutational experiments are shown for (**A**) HC4319, (**B**) DV1, (**C**) DV1 dimer, (**D**) DV3, (**E**) V1, and (**F**) vMIP-II. Residues are highlighted in red meaning their mutations resulting in a decrease in antagonist binding of over 60%, in purple meaning a decrease between 30% and 60%, in blue meaning a decrease between 10% and 29%, in yellow meaning no binding decrease. The results were averages of at least three independent experiments.

We conducted mutational analyses of the CXCR4 binding sites of HC4319, DV1, DV1 dimer, and DV3. All of these D-peptide antagonists displayed obvious reduction in binding to CXCR4 mutants Y45A, F87A, W94A, D97A, H113A, Y116A, N119A, D262N, D262E, E288Q, E288A, and E288D (Table 2), suggesting common or overlapping receptor binding sites of these D-peptides. On the other hand, differences among them were also observed. For example, the mutation of residue T117 on CXCR4 only affected the binding of DV1 dimer but not the other three D-peptides. The mutation of D171 caused decrease in receptor binding of DV1 dimer and DV3, but not HC4319 and DV1 while the mutation of D193 had effect on only HC4319 and DV1 dimer. Alanine substitution of residue R30 on CXCR4 did not affect the binding activity of all of eleven antagonists including D-peptides. Mutations at residues W161, W195, Q200, H203, Y255, I259 and H281 on CXCR4 had differential effects on D-peptides. For example, in comparing HC4319 and DV1, residues W161, Q200, H203, and I259 of CXCR4 are important for only DV1 whereas D193 and Y255 are important for HC4319 (Figure 5A,B and Table 2). The comparison of the important receptor binding sites of these D-peptides showed that they interact with CXCR4’s major and minor pockets in a similar manner overall with some variations on the degrees of importance of certain residues/sites (Figure 5A–D).

### The CXCR4 binding sites of linear L-peptide V1 and natural chemokine vMIP-II

In the co-crystal structure of the CXCR4-vMIP-II complex, the N-terminal residues 1-16 of vMIP-II recognize both the ligand binding pocket and N-terminus of CXCR4 [22]. The linear L-peptide V1 contains 21 amino acids derived from the N-terminus of vMIP-II. Thus, it is reasonable to anticipate that V1 may interact with CXCR4 in a manner similar to that of the N-terminus of vMIP-II. For this reason, we put these two antagonists in the same group for our analysis here. The mutational study showed that CXCR4 mutants F87A, W94A, Y116A and N119A displayed a marked decrease in the binding of V1 and vMIP-II while mutants D97A, D193A, and W195A showed a more modest effect (Table 2). In addition, mutations of Y45A, W161A, D262N, H281A, E288A and E288D exerted different effects on the binding of V1 and vMIP-II. Overall, the comparison of the CXCR4 binding sites of V1 and vMIP-II revealed their similar binding modes (Figure 5E,F). From the above analyses of the CXCR4 binding sites of D-peptides and L-peptide V1 and vMIP-II, it was evident that these two groups of antagonists displayed distinctive features in their receptor binding mechanisms. For example, mutations H113A and E288A had obvious effect on D-peptide binding but less or no effect on V1 and vMIP-II (Table 2), thus suggesting a more important role of these residues/sites for D-peptides. On the other hand, because D-peptides HC4319, DV1, DV1 dimer and DV3 and L-peptide V1 all contain residues derived from the N-terminus of vMIP-II and that these D-peptides and V1 are counterparts in some way with the same amino acids but different D- or L-configuration, it was not surprising that they also showed many common or overlapping binding residues/sites on CXCR4 as revealed by the similar effects on their binding of a number of CXCR4 point mutations.

### The CXCR4 binding sites of cyclic peptides CVX15 and LY2510924

**Figure 6:**
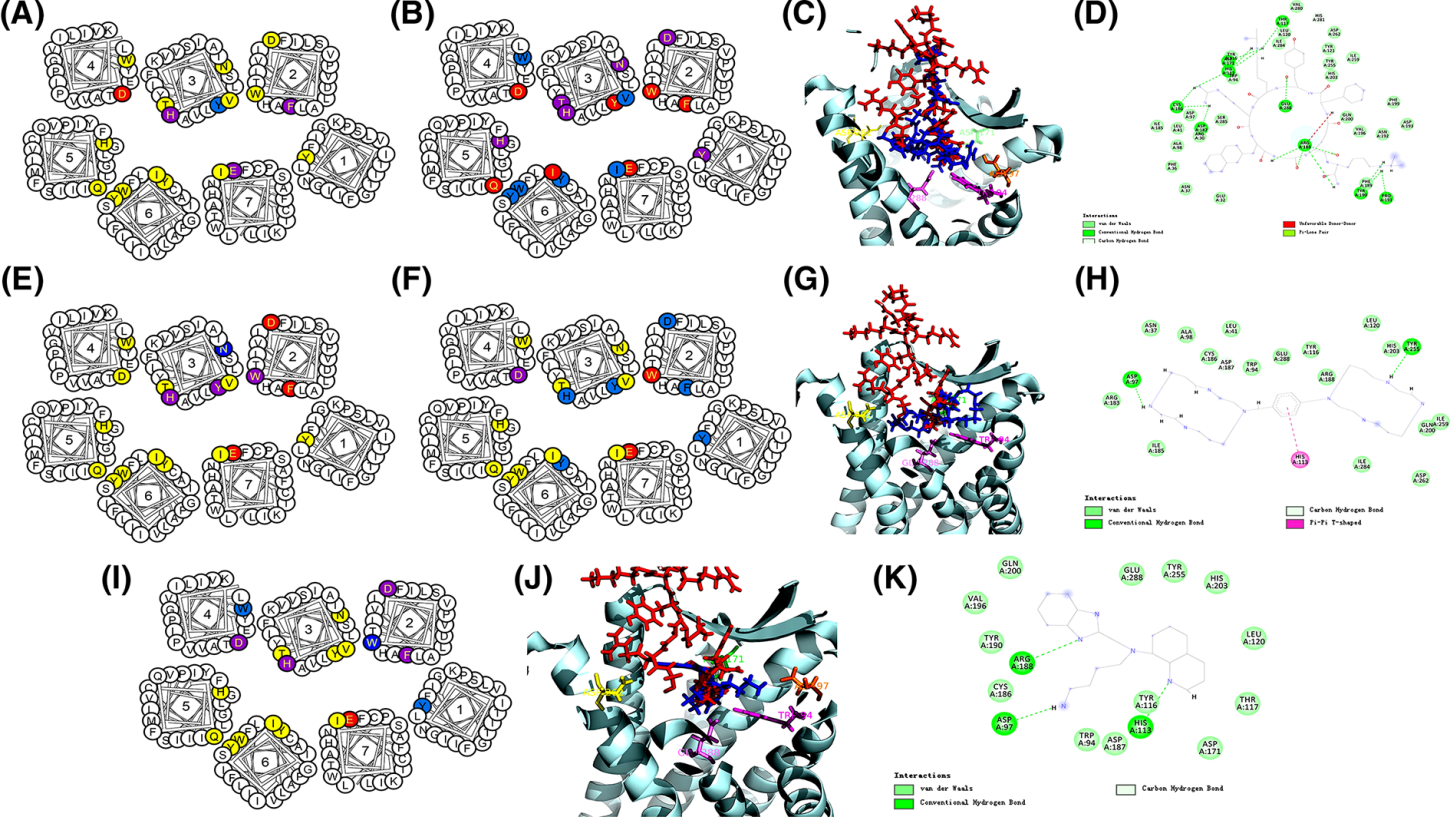
The CXCR4 binding sites of cyclic peptides and small molecules The residues implicated in antagonist binding as determined from mutational experiments are shown for (**A**) CVX15, (**B**) LY2510924, (**E**) IT1t, (**F**) AMD3100, and (**I**) AMD11070. The residues are colored in the same way and meaning as described above in Figure 5. Molecular docking calculations were performed for LY2510924, AMD3100 and AMD11070 to predict the CXCR4-antagonist complex structures. Using the co-crystal structure of CXCR4-CVX15 as a reference, the superposition of each of the above-mentioned antagonists (shown in blue) with CVX15 (red) is shown for (**C**) LY2510924, (**G**) AMD3100, and (**J**) AMD11070. The interactions of LY2510924, AMD3100, and AMD11070 with CXCR4 residues based on the docking results are illustrated for (**D**) LY2510924, (**H**) AMD3100, and (**K**) AMD11070. The molecular docking calculation was conducted using the crystal structure 3OE0 and the SYBYL program and displayed by PyMOL. The CXCR4 structure (in pale cyan) is shown in ribbons with important residues highlighted in colors: D262 (yellow), E288 (Violet), W94 (Magenta), D171 (Green), and D97 (Orange). The results were averages of at least three independent experiments.

In the co-crystal structure of the CXCR4-CVX15 complex, CVX15 occupies the major pocket of CXCR4, interacting with D193, D171 and D262 and forming H-bonds with H113 and a salt bridge with D262 of CXCR4 [23]. Our results with mutants F87A, D171A, D262N/E and E288Q showed significant reduction in CVX15 binding (Table 2 and Figure 6A), which is consistent with the important roles of these sites according to the co-crystal structure. All other mutations, except for Y116A having some effect, did not affect the receptor binding of CVX15. Another cyclic peptide examined here, LY2510924 is being used in combination with other drugs in clinical trials to treat various diseases [29,30,42,56]. Our mutational data showed that the binding affinity of LY2510924 was markedly decreased by the mutagenesis of CXCR4 residues Y45, F87, W94, D97, H113, Y116, T117, N119, D171, Q200, H203, I259, and E288 while alanine substitutions of V112, W161, W195, W252, Y255, Y256, H281, and I284 and mutant D262E also showed some reduction effect on ligand binding (Table 2 and Figure 6B).

In comparison with the linear D-peptides and L-peptide V1 described above which presumably have unconstrained and flexible conformations, cyclic peptide LY2510924 is expected to be more conformationally restrained due to the cyclization and thus more amenable to molecular docking simulation of their conformations when bound to CXCR4. As such, we attempted computer-aided docking calculation on LY2510924 in complex with CXCR4 guided by the mutational results described above. The predicted receptor bound conformation of LY2510924 in comparison with another cyclic peptide CVX15 with known CXCR4 bound crystal structure is shown in Figure 6C,D.

### The CXCR4 binding sites of small molecules IT1t, AMD3100, and AMD11070

In the co-crystal structure of CXCR4 with the small molecule antagonist IT1t [23], IT1t has salt bridge interactions with CXCR4 residues D97 and E288 and hydrophobic interaction with W94. Consistent with this structural observation, mutations at W94, D97, H113, Y116, and E288 had significant effect on IT1t binding (Table 2 and Figure 6E). The mutational effect of H113 is also in line with the results from ligand-pocket atomic contact contribution analysis of residues published previously by others [57]. Overall, the mutational analysis of IT1t here in this study is consistent with our previous computational analysis of this small molecule’s recognition of CXCR4 [58].

The small molecule CXCR4 antagonist, AMD3100 is the only clinically approved drug among the eleven antagonists analyzed in this study. It was previously reported by others that the interaction of AMD3100 with CXCR4 is largely attributed to electrostatic interactions between the cyclam rings of this small molecule with D171, D262 and E288 on the receptor [52–54]. In accordance with this notion, our mutational data showed that mutations of D171, D262 and E288 resulted in a large decrease in AMD3100 binding with CXCR4 (Table 2 and Figure 6F). Besides the CXCR4 residues previously known to be important for AMD3100 binding, the mutation of W94 resulted in the loss of ligand binding while mutations of Y45, F87, D97, H113, Y116, D193, and Y255 led to significant reduction in receptor binding of AMD3100. These new findings made in this study indicated the importance of these residues for AMD3100 interaction with CXCR4. Based on these mutational results, molecular docking calculation of AMD3100–CXCR4 interaction was conducted to predict the receptor–ligand complex structure using a crystal structure (PDB code: 3OE0) (Figure 6G,H). In the predicted binding pose, AMD3100 interacted with CXCR4 through the hydrophobic interaction of its positively charged cyclam rings with the side chain of W94 and electrostatic interaction with negatively charged side chain of E288 on CXCR4. Mutations of W94 and E288 would affect these interactions and account for the decrease in AMD3100 binding to these two CXCR4 mutants. Besides the residues of the trans-membrane domains of CXCR4, the residues in the ECLs may also be important for the binding of AMD3100 (Figure 6G,H).

**Figure 7:**
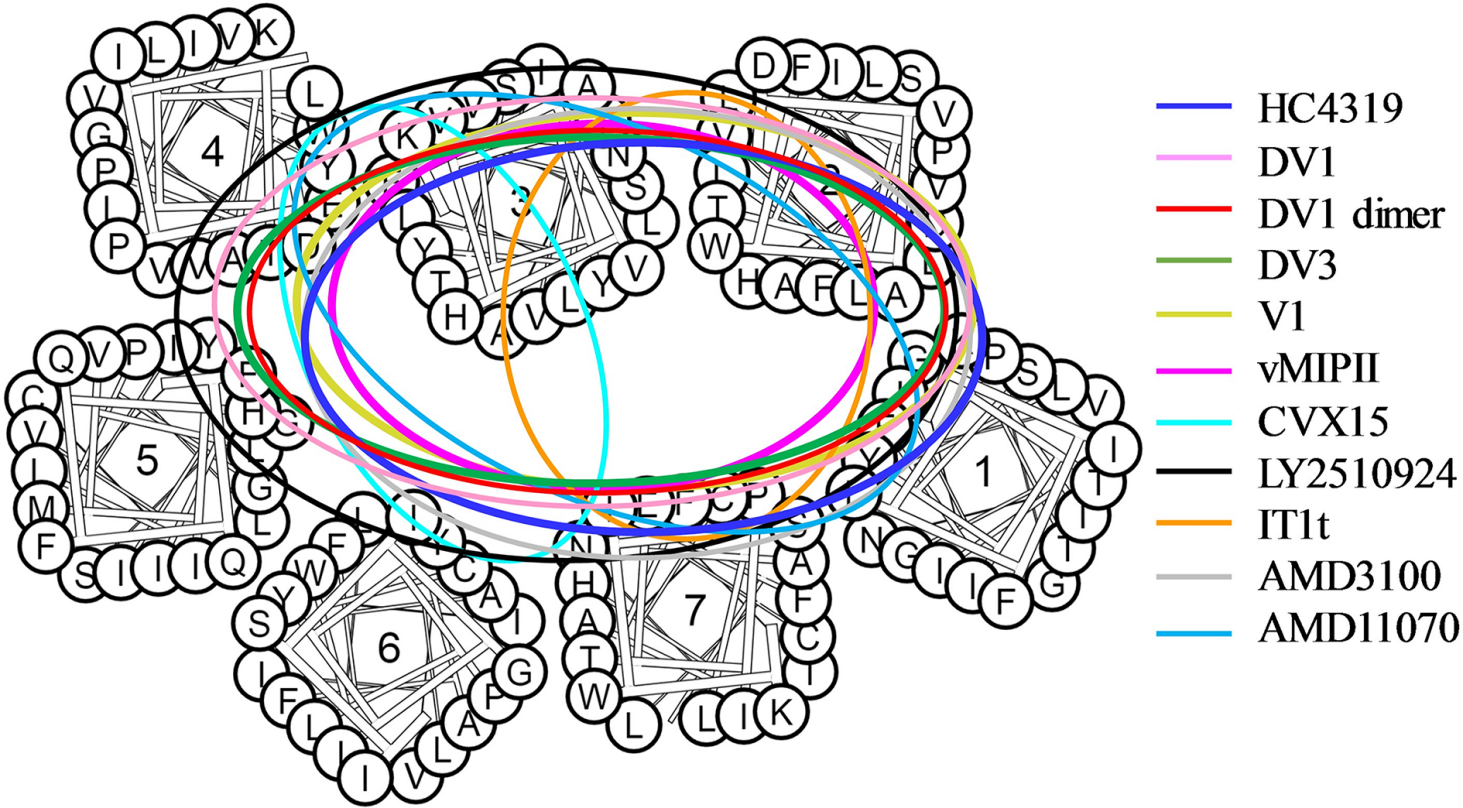
Comparison of the ligand's binding areas on the CXCR4 receptor for the eleven antagonists Binding areas of the antagonists are shown by circles or ovals in different colors of deep blue for HC4319, pink for DV1, red for DV1 dimer, green for DV3, yellow for V1, magenta for vMIP-II, cyan for CVX15, black for LY2510924, orange for IT1t, gray for AMD3100, and deep sky blue for AMD11070. The CXCR4 helices in Figures 4–7 were generated and downloaded from GPCRDB.

For AMD11070, previous molecular modeling studies have suggested the importance of CXCR4 residues Y45, W94, D97, D171, and E288 for the molecular interactions [59–61]. In this present study, our mutational data showed that point mutations of F87, D97, H113, D171, and E288 reduced the binding of AMD11070 while mutations of Y45, W94, W161, and W195 also showed an effect even though the effect was to a lesser extent (Table 2 and Figure 6I). For mutational analyses of AMD11070, we found new residues of CXCR4 that interacted with AMD11070, such as H113 and W161. We also conducted molecular docking to explore the possible binding pose to CXCR4 under the guidance of our mutational data. Molecular docking based on the published co-crystal structure (PDB code: 3OE0) suggested that the binding mode of AMD11070 is similar to that of IT1t (Figure 6J,K) [62]. Our mutational and docking analysis results are in general consistent with those published before by others except for the mutation of residue D262 that had no effect on the binding affinity of AMD11070 to CXCR4 [50]. To illustrate the overall features of the binding modes of these diverse CXCR4 antagonists, we showed the general comparison of their binding areas on CXCR4 (Figure 7), which served to highlight the common features in ligand–receptor binding.

### Design of new DV3 analogs with significantly enhanced CXCR4 binding

From the above described studies of 11 CXCR4 antagonists, we found overlapped residues on CXCR4 important for binding of most of these molecules that included W94, D97, H113, D171, D262, and E288, most of which are negatively charged residues. This observation prompted us to hypothesize that introducing more positive charge into the ligand may further strengthen ligand interaction with the important negative charge of the receptor. To test this hypothesis, we designed new analogs of DV3, a representative D-peptide antagonist of CXCR4 studied above by incorporating positively charged Arg residues aimed at promoting charge–charge interactions with the receptor. It is noted that a number of reported CXCR4 antagonists contain positively residues including T22 and its derivatives, tachyplesin and polyphemusin derived peptides [63,64], cyclic peptide antagonist FC131 containing the Arg-Arg-2-Nal (D-3-(2-naphthyl)-alanine) tripeptide motif [65], and arginine-based peptide ALX40-4C [66]. Here, in this study, we modified DV3 in accordance with positively charged lysine and arginine and pharmacophore of T22 and its analogues (Table 1). N terminal modification of DV3 (HM60303) significantly reduced the binding affinity (IC_50_ > 50 μM) whereas C terminal modification increased the binding affinity. New DV3 analogs with 1-2 arginine residues or Nal-Arg-Arg added to the peptide sequence displayed much stronger CXCR4 binding than DV3 with an IC_50_ values from 377.4 nM (HM13) to 706.6 nM (HM60323). Interestingly, HM70116 containing amino acids 1–7 of DV3 plus the D-sequence of Nal-Arg-Arg showed the strongest binding among these analogs, with an IC_50_ of 193.9 nM. To further assess the CXCR4 binding selectivity of these new analogs, we examined whether HM70116, the most potent and representative analog, could bind another chemokine receptor CXCR7 since CXCR7 and CXCR4 share a natural ligand SDF-1α. HM701116 showed only marginal competitive binding to CXCR7 even at a high concentration (20% binding at 10 μM) in contrast with its nanomolar binding to CXCR4 described above (Supplementary Figure S2). This demonstrates the peptide’s selective binding toward the targeted receptor CXCR4.

### The inhibitory effect of HM70116 on CXCR4-mediated intracellular calcium mobilization, cell migration and CXCR4 internalization

**Figure 8:**
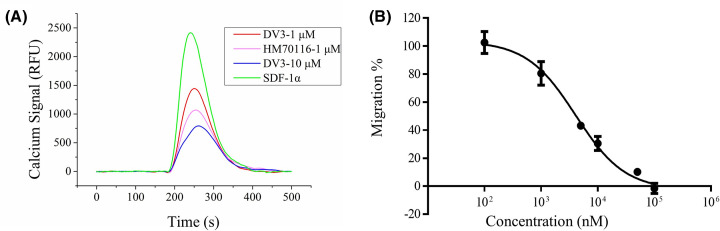
Inhibitory effects of HM70116 on SDF-1α-induced calcium mobilization and cell migration (**A**) The inhibition of HM70116 on intracellular calcium increase. (**B**) The inhibition of HM70116 cell migration. The results were averages of at least three independent experiments.

**Figure 9:**
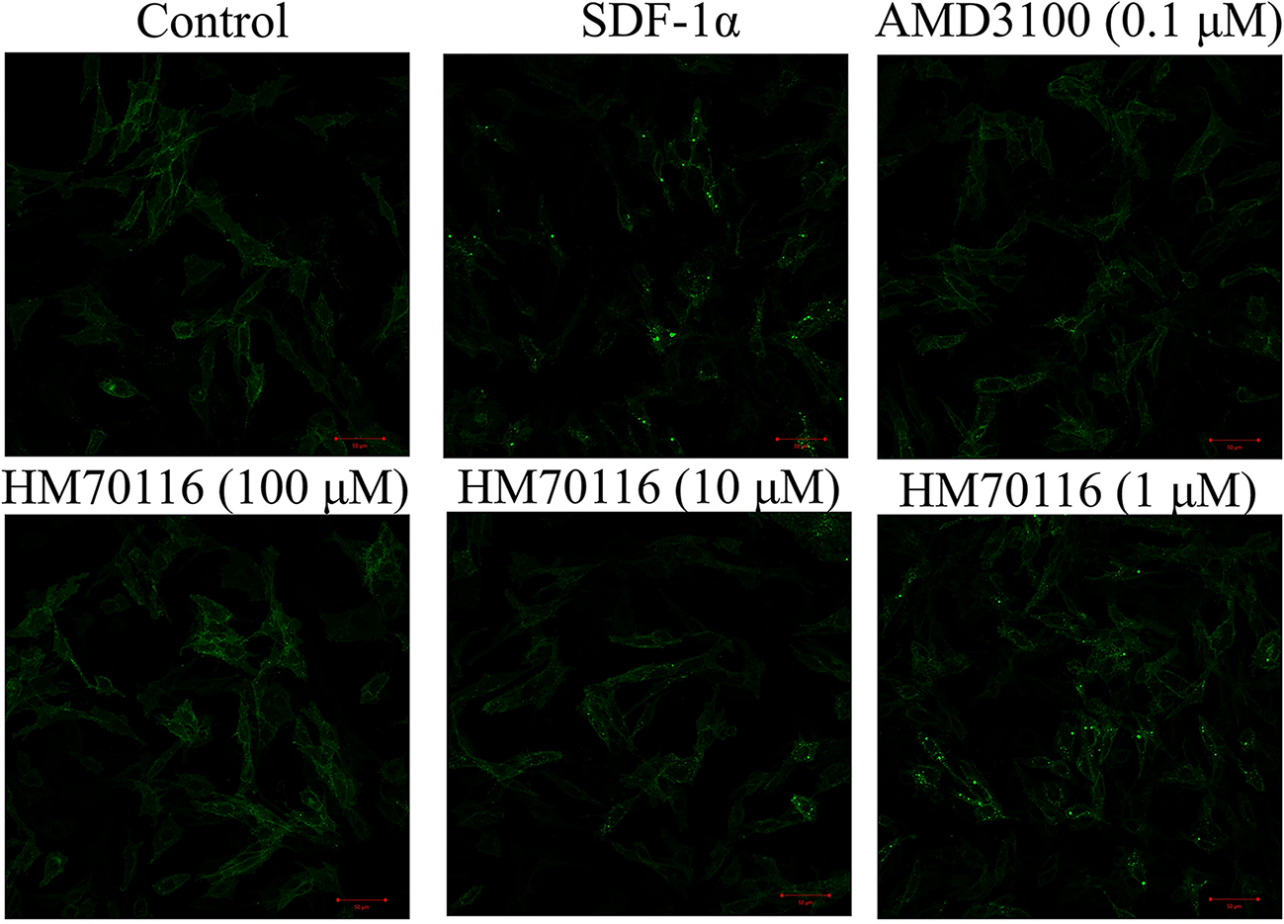
Inhibitory effects of HM70116 on SDF-1α-induced CXCR4 internalization The inhibition of HM70116 on CXCR4 internalization and the concentrations used are shown (scale bar: 50 μm). The results were representative of at least three independent experiments.

HM70116 was further studied in its biological effect on CXCR4-mediated cellular functions and found to be potent in antagonizing SDF-1α-CXCR4-mediated intracellular calcium mobilization, cell migration and CXCR4 internalization (Figures 8 and 9). SupT1 cells pretreated with HM70116 (1 μM) potently inhibited intracellular calcium mobilization (Figure 8A). HM70116 antagonized CXCR4-mediated cell migration in a dose-dependent manner with an IC_50_ of 4.8 ± 1.5 μM (Figure 8B). HM70116 effectively prevented CXCR4 internalization at 100 and 10 μM (Figure 9).

### The CXCR4 binding site characterization of HM70116

**Figure 10:**
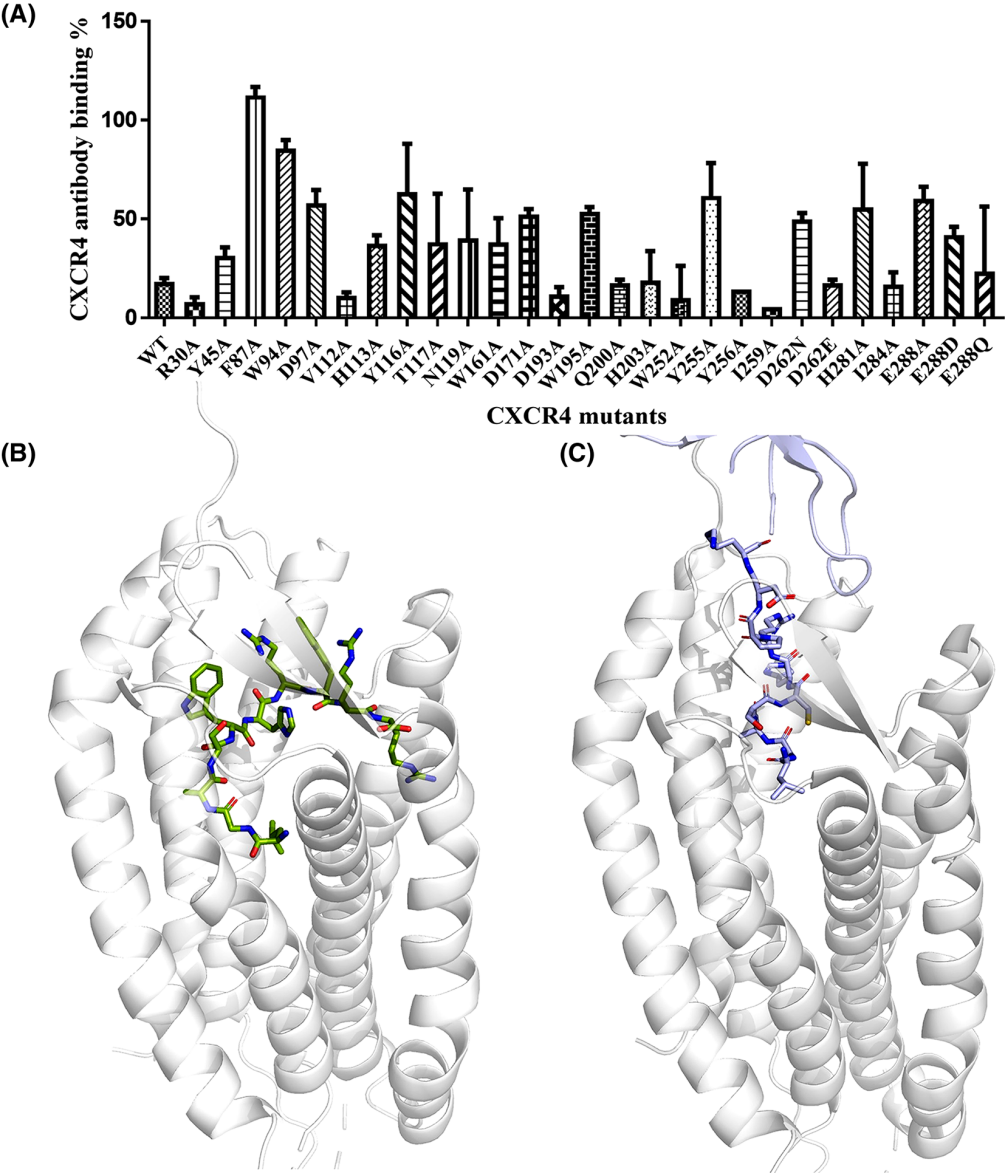
The mutational analysis and molecular modeling of HM70116 interaction with CXCR4 (**A**) Mutational analysis of HM70116 interaction with CXCR4 binding pocket. The data was averaged from three independent experiments with duplicate values. The data were shown as mean ± standard error of the mean (S.E.M.). (**B**) MD simulation of HM70116-CXCR4 complex. (**C**) Binding mode of the N-terminus of vMIP-II in the CXCR4-binding pocket.

To further characterize the molecular mechanism of action of HM70116, we conducted mutational analysis of its binding interaction with CXCR4 following the method described above for 11 CXCR4 antagonists and compared the results with those for the parent antagonist DV3. Reduction in binding to CXCR4 mutants of F87A, W94A, Y116A, T117A, D171A, W195A, Y255A, D262N, and H281A of HM70116 is greater than DV3 (Figure 10A), while only mutant H113A affected DV3 more than its analog HM70116 (Table 2). This indicates that HM70116 has more extensive and stronger interaction with CXCR4-binding pocket than DV3 which is consistent with the significantly increased receptor binding of HM70116. Molecular dynamics simulation also revealed that the binding pose of HM70116 (Figure 10B) is quite different from that of the N-terminal 1–10 residues of vMIP-II (Figure 10C) of which sequence is DV3 derived.

## Discussion

Since the discovery of the CXCR4 chemokine receptor as one of the two principal co-receptors for HIV-1 entry in 1996 [10], CXCR4 has been a target of intensive biological, pharmaceutical and clinical research of its roles in not only HIV-1 infection but a growing number of pathologies and medical applications. The research of CXCR4 also resulted in the development of diverse types of peptide-based and non-peptidic small molecule antagonists of this receptor including one drug currently used clinically and many others in the clinical trial and preclinical development stages [18,67]. As these antagonists were developed and reported by different groups and companies, there is insufficient information about the comparison of their cellular activities and molecular mechanisms of receptor interactions. Here in this study, we conducted comparative cell-based biological assays on a panel of 11 different molecules representative of the diverse types of CXCR4 antagonists reported in the literature by others and us. These cell-based assays were well established and used by us and others and included competitive receptor binding, calcium influx, cell migration, and receptor internalization. The results provide an objective comparison of the cellular biological activities of these different antagonistic molecules under the same assay systems and conditions, which may be a helpful reference for future developmental efforts on these agents. From these studies, we found that, for the D-peptide series, dimeric D-peptides such as HC4319 and DV1 dimer were much more potent CXCR4 binders than monomeric D-peptides like DV1 and DV3, indicating the advantage of bivalent ligands. For other peptides and small molecules, we found that a cyclic peptide CVX15 and a small molecule AMD11070 displayed very high receptor affinity with IC_50_ values comparable to that of wild-type chemokines such as vMIP-II. AMD11070, an optimized analog of AMD3100, showed much enhanced CXCR4 binding which was consistent with that reported in the literature. For some antagonists, their binding activity as measured by IC_50_ values in this study was slightly less potent than the published data as discussed in the introduction, possibly due to the use of overly expressed cell line in our study, different numbers of cells, and different competitor molecules (i.e., ^125^I-labeled SDF-1α versus 12G5 antibody in our study). LY2510924 showed much lower potency than that published, partly for the reasons mentioned above, and that they used a mix of ^125^I-labeled SDF-1α and unlabeled SDF-1α in the binding assay [30]. As to the cell-based functional assays measuring the inhibitory activities of these antagonists on SDF-1α-CXCR4-mediated cellular signaling and functions, we found that dimeric D-peptides HC4319 and DV1 dimer were more potent in blocking calcium influx and cell migration than monomeric D-peptides while CVX15, IT1t, and AMD11070 were very effective in antagonizing cellular signaling and migration. It was seen that the inhibitory activities of these antagonists on cell-based functions, even though measured at only one chosen concentration, were in general consistent with their different CXCR4 competitive binding IC_50_ values. This suggests that quantitative dose-dependent evaluation of an antagonist’s receptor competitive binding IC_50_ can guide qualitative comparative assessment of different antagonists’ activities in cellular functional assays to some extent, especially for molecules with similar structures.

In addition to comparing the receptor binding and cellular functional activities as discussed above, we performed CXCR4 site-directed mutagenesis experiments to determine the receptor binding sites of each of these eleven antagonists on the TM domains of the receptor. This allowed us to evaluate and compare the common as well as distinctive receptor binding modes of these different antagonistic molecules. Among the many CXCR4 TM domain point mutations examined here, it was noteworthy that two particular mutations, F87A and E288Q significantly affected the receptor binding across all 11 antagonists (Table 2). The residue F87, which is buried in protein hydrophobic cores shown in the crystal structure of CXCR4, seems to play a critical role in ligand recognition and/or stabilizing the structural integrity of the receptor which in either case can affect directly or indirectly the binding of antagonistic ligands. This notion was suggested in a recent study where F87, W252 and L120 were thought to form a hydrophobic triad important for the structure maintenance and signal transduction [68]. It was also suggested that F87 may play a general role in the GPCR superfamily due to its high conservation observed among GPCRs [68]. As to E288, its role was revealed in a previous comprehensive study by others of CXCR4 TM domains where 41 residues were found to be involved in SDF-1α-CXCR4 signaling, including D97, D187, F189, H281, and D262 important for ligand binding, and W94, Y45, Y116, and E288 for signaling [16]. Many of these residues were included in our mutational analyses of this present study and the significant effect of the E288Q mutation for all 11 antagonists was consistent with the other study described above.

The two residues F87 and E288 discussed above serve as the focal points to illustrate the common or overlapping binding modes across structurally highly diverse antagonists. On the other hand, examination of other point mutations, except for R30A which had no effect for all antagonists studied here, suggested different roles of these TM residues for CXCR4 binding of different antagonistic molecules, thus revealing the distinctive features in their receptor binding modes. The common, overlapping as well as distinctive CXCR4-binding sites of the eleven antagonists are schematically illustrated to provide a general comparison of the molecule interactions between these molecules and the receptor (Figure 7). From this, it can be seen that D-peptides (HC4319, DV1, DV1 dimer and DV3) and L-peptide V1 containing the same sequence fragment from the N-terminus of vMIP-II have quite similar CXCR4 binding sites despite the different D- and L-configurations and that the binding modes of small molecules AMD11070 and AMD3100 appear to be more similar to another small molecule IT1t than to the cyclic peptide CVX15. The receptor binding of AMD3100 was affected by W94 and D97 mutations in the minor subpocket and D171 and D262 mutations in the major subpocket of CXCR4, suggesting that AMD3100 interacts with both subpockets and that the binding pose of AMD3100 might be quite different from the previous report that AMD3100 is mainly bound to the major subpocket of CXCR4 [69]. We tend to favor the model that one cyclam of AMD3100 interacts with the minor subpocket of CXCR4 while AMD3100’s another cyclam ring interacts with the major subpocket of CXCR4 which is similar to another previously reported binding mode (or the second binding mode of AMD3100) [50]. In our mutational analyses of AMD11070, its receptor binding was mainly affected by the residues located in the minor subpocket of CXCR4 and mutation of D171 in the major subpocket. Our molecular docking results seemed to agree well with the mutational data in that the binding pocket of AMD11070 overlapped with that of IT1t and that the contact area was similar to what was previously reported [50,69]. It is noteworthy that the receptor binding of cyclic peptide LY2510924, currently in clinical trials as a drug candidate, was affected by most point mutations among all the antagonists tested in this study. This tends to imply that LY2510924 has more extensive contacts with the receptor than other antagonists.

To further investigate the observation of a number of negatively charged residues on CXCR4 found to be involved in binding to many of 11 antagonists studied here, we designed and synthesized a series of new analogs of DV3, a representative D-peptide antagonist, containing positively charged arginine residues aimed at interacting with the above mentioned negatively charged residues on the receptor. These new analogs displayed enhanced receptor binding as expected. Among them, HM70116 showed the greatest increase in CXCR4 binding. Mutational analysis revealed that, in the motif of Arg1-Arg2-Nal3 introduced in this molecule, Arg2 interacts with residues H113 and D171, Nal3 interacts with TM5 of CXCR4 through the hydrophobic pocket, and Arg1 interacts with the D187 of the extracellular 2 of CXCR4 [70].

In conclusion, using cell-based assays, site-directed mutagenesis and molecular modeling techniques, we carried out direct comparative analyses of the cellular activities and molecular interactions of a panel of eleven CXCR4 antagonists selected from diverse structural classes and molecular sizes. These studies provide comparison of the bioactivities and binding mechanisms of these molecular agents either currently used clinically or under preclinical or clinical development. The residues or sites implicated in ligand recognition as determined from our studies for each of these antagonists reveal both common and different features in their interactions with the CXCR4 receptor, which shed further insight into the mechanism of action of CXCR4-targeted agents. As CXCR4 is involved with a growing number of human diseases and medical applications, the information obtained from the present study should augment the foundation for the further development of these existing antagonists as well as their new analogs shown in this study.

## Materials and methods

### Materials

Plasmid pcDNA3-CXCR4 was constructed as previously described [17,71,72]. The anti-CXCR4 monoclonal antibody (mAb) 12G5 was purchased from BD Bioscience (San Jose, CA, U.S.A.). SDF-1α was purchased from Prospec (Rehovot, Israel). SupT1 cell line was purchased from ATCC (Manassas, VA, U.S.A.). Chinese hamster ovary (CHO) cells were purchased from China Infrastructure of Cell Line Resources (Beijing, China). Dulbecco’s Modified Eagles Medium (DMEM) and RPMI 1640 (Corning, NY, U.S.A.) with 10% fetal bovine serum (Gibco, Grand Island, NY, U.S.A.) and 1% penicillin-streptomycin were used for cell culture. G418 SULFATE was purchased from Amresco (Solon, OH, U.S.A.). DV1, DV1 dimer, HC4319, and V1 were synthesized by GLBiochem (Shanghai, China). AMD3100 was purchased from C-aring (Wuhan, China). Recombinant vMIP-II was acquired from Peprotech (Rocky Hill, NJ, U.S.A.). AMD11070 was obtained from MedChemExpress (Monmouth Junction, NJ, U.S.A.). DV3 and IT1t were synthesized in our lab following previous publications [25,31]. HM70116 and other peptides were synthesized by GLBiochem. Q5® High-Fidelity DNA polymerase and DpnI were obtained from New England Biolabs (Beverly, MA, U.S.A.). Competent *Escherichia coli* was purchased from Biomed (Beijing, China). QIAGEN Plasmid Plus Midi Kit was purchased from Qiagen (Duesseldorf, Germany). Lipofectamine 2000 was obtained from Invitrogen (San Diego, CA, U.S.A.). Transwells were purchased from Corning Incorporated (Corning, NY, U.S.A.).

### Cell transfection

Plasmid pcDNA3-CXCR4 and mutated pcDNA3-CXCR4 were extracted and transfected into CHO cells using Lipofectamine 2000 according to the manufacturer’s guidelines. The selective medium containing G418 at a concentration of 1.2 mg/ml was applied to isolate stably transfected cells. CHO cells expressing wild-type CXCR4 and mutant CXCR4 were obtained using fluorescence-activated cell sorting (FACS).

### Calcium mobilization assay

SupT1 cells were collected and washed using HHBS buffer (Hanks’ Balanced Salt solution supplemented with 20 mM HEPES) and incubated with 1 mM probenecid (Energy Chemical, China) and 4 μM Fluo-4 (AAT bioquest, U.S.A.) for 37°C for 30 min with vortex every 5 min. After being washed with HHBS, cells were suspended in PBS at 2 × 10^6^/ml and seeded 200 μl cells and various concentrations of compounds per well in 96-well plate. Following 50 nM SDF-1α was added to each well, the fluorescence intensity was measured (*E*x/*E*m = 496/516 nm) using EnSpire® multimode plate reader (PerkinElmer, U.S.A.).

### Cell migration assay

SupT1 cells (2 × 10^6^ cells per well in 75 μl) were collected and washed using assay buffer (RPMI 1640 supplemented with 0.5% BSA) and incubated with various concentrations of tested compounds for 30 min in the upper well of the transwell-96 well plates with 5.0 µm pore polycarbonate membrane insert (Corning, U.S.A.). Then, 200 μl assay buffer containing 2 nM SDF-1α was added to the lower well and cells were allowed to migrate for 3 h. Migrated cells were measured using celltiter-96 reagent. The data were displayed as mean ± standard deviation (S.D.).

### CXCR4-mediated internalization assay

CXCR4 and EGFP were amplified using a primer shown in Supplementary Table S1. The pcDNA3 vector was digested by restriction-enzyme EcoR1 and Apa1. CXCR4 was digested by restriction-enzyme EcoR1 and Xba1. EGFP was digested by restriction-enzyme Xba1 and Apa1. CXCR4–EGFP conjugate was constructed by ligation of three DNA fragments using T4 ligase. The vector containing CXCR4 ligated EGFP at the C-terminus was transfected and expressed in CHO cells in 100 μl DMEM medium supplemented with 2% FBS at 1 × 10^5^/ml which was seeded in 96-well black plate glass bottom and incubated at 37°C overnight. Following incubation with tested compounds for 10 min, 50 nM of SDF-1α was added to each well and the internalized CXCR4 was observed using a confocal microscopy (Zeiss LSM880).

### Site-directed mutagenesis of CXCR4

All of the CXCR4 mutants were prepared using PCR technology. Briefly, pairs of asymmetrical primers containing the desired amino acid mutations (Supplementary Tables S1 and S2), each complementary to opposite strands of the pcDNA3–CXCR4, were designed for amplification by Q5® High-Fidelity DNA polymerase and the generation of mutated constructs. The methylated pcDNA3–CXCR4 templates were digested by DpnI. The mutated pcDNA3–CXCR4 was transformed into competent *E. coli* TOP10 for long-term storage and amplification.

### Competitive binding of the antagonists to CXCR4 and its mutants

The procedure for the CXCR4 competitive binding experiments was published previously [27,73]. Briefly, CXCR4 antibody 12G5 (250 ng/ml) was incubated with 5 × 10^5^ cells and varying concentrations of unlabeled compounds in a final volume of 100 μl of assay buffer (PBS with 0.5% BSA and 0.05% NaN_3_) in 96-well plates for 40 min on ice, after being washed with assay buffer once. The cells were incubated with FITC-conjugated second antibody IgG for 30 min on ice, then washed with assay buffer three times. The fluorescence intensity was measured for excitation at 485 nm and emission at 535 nm using multimode plate reader (PerkinElmer). The IC_50_ values were calculated using graphpad Prism 7. Experiments were performed at least three times.

### Molecular modeling analyses of CXCR4-antagonist interactions

Molecular modeling was conducted using the SYBYL program (version 2.1.1) and the published co-crystal structures of CXCR4 with IT1t, CVX15 and vMIP-II. Before docking, the crystal structure (PDB code: 3OE0) of CXCR4 was prepared by removing the ligand and other molecules such as waters and fatty acids from the receptor complex. Hydrogens and charges were then added, followed by protonation and side-chain repair to complete the process. After that, the receptor’s pocket for antagonist docking was generated by protomol in the mode of ligand. The docking mode of surflex-dock was used for surface docking, and the extracted ligand was set for molecular reference. Energy minimization of the ligand was performed, and hydrogens were added before surflex docking.

### Molecular modeling of HM70116–CXCR4 interaction

The extended structure of HM70116 was built with PyMoL and relaxed with ROSETTA3 to optimize the energy and conformation [74–76]. The crystal structure of vMIP-II–CXCR4 complex (PDB: 4RWS) was selected and also relaxed for molecular docking. The starting structure of HM70116–CXCR4 for molecular docking was generated by aligning HM70116 with vMIP-II in the vMIP-II–CXCR4 complex using PyMOL, since HM70116 and vMIP-II have similar N-terminal sequences. Molecular docking of HM70116 to CXCR4 was performed using ROESTTA3 Flexpepdock application [77,78]. Briefly, the starting structure of HM70116–CXCR4 was refined first with Flexpepdock and then subjected to molecular docking. To effectively sample the conformational space, the number of both outer cycles of each simulation and inner-cycles for both rigid-body and torsion-angle Monte Carlo minimization was set to 12. Furthermore, to produce more accurate models, we generated ROSETTA recognizable constraint file based on our experimental results of mutation mapping and used it in docking calculation. The 3000 independent Flexpepdock outputs were generated and scored. Finally, all of the outputs were rescored by summing the total score, I_sc and pep_sc terms and reordering. Top 20 models were selected to cluster and analyze. Visual analyzing outputs and generating 3D structure representations were finished with PyMOL.

## Supplementary Material

Supplementary Figure S1-S2 and Tables S1-S3

## Data Availability

Molecular model was submitted to Protein Model Database has been assigned the following PMDB id: PM0084214[62]. All the data generated or analyzed during this study are included in this published article. All the raw data is available with the corresponding author on request.

## Open Access

Open access for this article was enabled through a transformative open access agreement between Portland Press and the University of California.

## Abbreviations

CCR5: C-C chemokine receptor type 5
CHO: chinese hamster ovary
CXCL12: C-X-C motif chemokine ligand 12
CXCR4: C-X-C chemokine receptor type 4
GPCR: G protein-coupled receptor
HIV-1: human immunodeficiency virus type 1
SDF-1α: stromal cell-derived factor-1 alpha
TM: transmembrane
vMIP-II: viral macrophage-inflammatory protein-II
WHIM syndrome: warts hypogammaglobulinemia, infections, and myelokathexis syndrome

## References

[1] Fredriksson R., Lagerstrom M.C., Lundin L.G. and Schioth H.B. (2003) The G-protein-coupled receptors in the human genome form five main families. Phylogenetic analysis, paralogon groups, and fingerprints. Mol. Pharmacol. 63, 1256–1272 10.1124/mol.63.6.1256 12761335

[2] Hauser A.S., Attwood M.M., Rask-Andersen M., Schioth H.B. and Gloriam D.E. (2017) Trends in GPCR drug discovery: new agents, targets and indications. Nat. Rev. Drug Discov. 16, 829–842 10.1038/nrd.2017.178 29075003 PMC6882681

[3] Teicher B.A. and Fricker S.P. (2010) CXCL12 (SDF-1)/CXCR4 pathway in cancer. Clin. Cancer Res. 16, 2927–2931 10.1158/1078-0432.CCR-09-2329 20484021

[4] Zou Y.R., Kottmann A.H., Kuroda M., Taniuchi I. and Littman D.R. (1998) Function of the chemokine receptor CXCR4 in haematopoiesis and in cerebellar development. Nature 393, 595–599 10.1038/31269 9634238

[5] McGrath K.E., Koniski A.D., Maltby K.M., McGann J.K. and Palis J. (1999) Embryonic expression and function of the chemokine SDF-1 and its receptor, CXCR4. Dev. Biol. 213, 442–456 10.1006/dbio.1999.9405 10479460

[6] Tachibana K., Hirota S., Iizasa H., Yoshida H., Kawabata K., Kataoka Y. et al. (1998) The chemokine receptor CXCR4 is essential for vascularization of the gastrointestinal tract. Nature 393, 591–594 10.1038/31261 9634237

[7] Hermann P.C., Huber S.L., Herrler T., Aicher A., Ellwart J.W., Guba M. et al. (2007) Distinct populations of cancer stem cells determine tumor growth and metastatic activity in human pancreatic cancer. Cell Stem Cell. 1, 313–323 10.1016/j.stem.2007.06.002 18371365

[8] Muller A., Homey B., Soto H., Ge N.F., Catron D., Buchanan M.E. et al. (2001) Involvement of chemokine receptors in breast cancer metastasis. Nature 410, 50–56 10.1038/35065016 11242036

[9] Orimo A., Gupta P.B., Sgroi D.C., Arenzana-Seisdedos F., Delaunay T., Naeem R. et al. (2005) Stromal fibroblasts present in invasive human breast carcinomas promote tumor growth and angiogenesis through elevated SDF-1/CXCL12 secretion. Cell 121, 335–348 10.1016/j.cell.2005.02.034 15882617

[10] Feng Y., Broder C.C., Kennedy P.E. and Berger E.A. (1996) HIV-1 entry cofactor: functional cDNA cloning of a seven-transmembrane, G protein-coupled receptor. Science 272, 872–877 10.1126/science.272.5263.872 8629022

[11] Zhang L., He T., Talal A., Wang G., Frankel S.S. and Ho D.D. (1998) In vivo distribution of the human immunodeficiency virus/simian immunodeficiency virus coreceptors: CXCR4, CCR3, and CCR5. J. Virol. 72, 5035–5045 10.1128/JVI.72.6.5035-5045.1998 9573273 PMC110066

[12] Esbjornsson J., Mansson F., Martinez-Arias W., Vincic E., Biague A.J., da Silva Z.J. et al. (2010) Frequent CXCR4 tropism of HIV-1 subtype A and CRF02_AG during late-stage disease–indication of an evolving epidemic in West Africa. Retrovirology 7, 23 10.1186/1742-4690-7-23 20307309 PMC2855529

[13] LaBonte J., Lebbos J. and Kirkpatrick P. (2003) Enfuvirtide. Nat. Rev. Drug Discov. 2, 345–346 10.1038/nrd1091 12755128

[14] Oberlin E., Amara A., Bachelerie F., Bessia C., Virelizier J.L., Arenzana-Seisdedos F. et al. (1996) The CXC chemokine SDF-1 is the ligand for LESTR/fusin and prevents infection by T-cell-line-adapted HIV-1. Nature 382, 833–835 10.1038/382833a0 8752281

[15] Bleul C.C., Farzan M., Choe H., Parolin C., Clark-Lewis I., Sodroski J. et al. (1996) The lymphocyte chemoattractant SDF-1 is a ligand for LESTR/fusin and blocks HIV-1 entry. Nature 382, 829–833 10.1038/382829a0 8752280

[16] Wescott M.P., Kufareva I., Paes C., Goodman J.R., Thaker Y., Puffer B.A. et al. (2016) Signal transmission through the CXC chemokine receptor 4 (CXCR4) transmembrane helices. Proc. Natl. Acad. Sci. U.S.A. 113, 9928–9933 10.1073/pnas.1601278113 27543332 PMC5024644

[17] Choi W.T., Tian S., Dong C.Z., Kumar S., Liu D., Madani N. et al. (2005) Unique ligand binding sites on CXCR4 probed by a chemical biology approach: implications for the design of selective human immunodeficiency virus type 1 inhibitors. J. Virol. 79, 15398–15404 10.1128/JVI.79.24.15398-15404.2005 16306611 PMC1316031

[18] Choi W.-T., Duggineni S., Xu Y., Huang Z. and An J. (2012) Drug discovery research targeting the CXC chemokine receptor 4 (CXCR4). J. Med. Chem. 55, 977–994 10.1021/jm200568c 22085380 PMC3476736

[19] Klasen C., Ohl K., Sternkopf M., Shachar I., Schmitz C., Heussen N. et al. (2014) MIF promotes B cell chemotaxis through the receptors CXCR4 and CD74 and ZAP-70 signaling. J. Immunol. 192, 5273–5284 10.4049/jimmunol.1302209 24760155

[20] Bernhagen J., Krohn R., Lue H., Gregory J.L., Zernecke A., Koenen R.R. et al. (2007) MIF is a noncognate ligand of CXC chemokine receptors in inflammatory and atherogenic cell recruitment. Nat. Med. 13, 587–596 10.1038/nm1567 17435771

[21] Arimont M., Sun S.L., Leurs R., Smit M., de Esch I.J.P. and de Graaf C. (2017) Structural analysis of chemokine receptor-ligand interactions. J. Med. Chem. 60, 4735–4779 10.1021/acs.jmedchem.6b01309 28165741 PMC5483895

[22] Qin L., Kufareva I., Holden L.G., Wang C., Zheng Y., Zhao C. et al. (2015) Structural biology. Crystal structure of the chemokine receptor CXCR4 in complex with a viral chemokine. Science 347, 1117–1122 10.1126/science.1261064 25612609 PMC4362693

[23] Wu B., Chien E.Y., Mol C.D., Fenalti G., Liu W., Katritch V. et al. (2010) Structures of the CXCR4 chemokine GPCR with small-molecule and cyclic peptide antagonists. Science 330, 1066–1071 10.1126/science.1194396 20929726 PMC3074590

[24] Zhou N.M., Luo Z.W., Luo J.S., Hall J.W. and Huang Z.W. (2000) A novel peptide antagonist of CXCR4 derived from the N-terminus of viral chemokine vMIP-II. Biochemistry 39, 3782–3787 10.1021/bi992750v 10736178

[25] Zhou N.M., Luo Z.W., Luo J.S., Fan X.J., Cayabyab M., Hiraoka M. et al. (2002) Exploring the stereochemistry of CXCR4-peptide recognition and inhibiting HIV-1 entry with D-peptides derived from chemokines. J. Biol. Chem. 277, 17476–17485 10.1074/jbc.M202063200 11880384

[26] Xu Y., Duggineni S., Espitia S., Richman D.D., An J. and Huang Z. (2013) A synthetic bivalent ligand of CXCR4 inhibits HIV infection. Biochem. Biophys. Res. Commun. 435, 646–650 10.1016/j.bbrc.2013.05.038 23688427 PMC3752463

[27] Choi W.T., Kumar S., Madani N., Han X., Tian S., Dong C.Z. et al. (2012) A novel synthetic bivalent ligand to probe chemokine receptor CXCR4 dimerization and inhibit HIV-1 entry. Biochemistry 51, 7078–7086 10.1021/bi2016712 22897429 PMC3476724

[28] Kledal T.N., Rosenkilde M.M., Coulin F., Simmons G., Johnsen A.H., Alouani S. et al. (1997) A broad-spectrum chemokine antagonist encoded by Kaposi's sarcoma-associated herpesvirus. Science 277, 1656–1659 10.1126/science.277.5332.1656 9287217

[29] Galsky M.D., Vogelzang N.J., Conkling P., Raddad E., Polzer J., Roberson S. et al. (2014) A phase I trial of LY2510924, a CXCR4 peptide antagonist, in patients with advanced cancer. Clin. Cancer Res. 20, 3581–3588 10.1158/1078-0432.CCR-13-2686 24727324

[30] Peng S.B., Zhang X., Paul D., Kays L.M., Gough W., Stewart J. et al. (2015) Identification of LY2510924, a novel cyclic peptide CXCR4 antagonist that exhibits antitumor activities in solid tumor and breast cancer metastatic models. Mol. Cancer Ther. 14, 480–490 10.1158/1535-7163.MCT-14-0850 25504752

[31] Thoma G., Streiff M.B., Kovarik J., Glickman F., Wagner T., Beerli C. et al. (2008) Orally bioavailable isothioureas block function of the chemokine receptor CXCR4 in vitro and in vivo. J. Med. Chem. 51, 7915–7920 10.1021/jm801065q 19053768

[32] Donzella G.A., Schols D., Lin S.W., Este J.A., Nagashima K.A., Maddon P.J. et al. (1998) AMD3100, a small molecule inhibitor of HIV-1 entry via the CXCR4 co-receptor. Nat. Med. 4, 72–77 10.1038/nm0198-072 9427609

[33] Schols D., Claes S., Hatse S., Princen K., Vermeire K., De Clercq E. et al. (2003) Anti-HIV activity profile of AMD070, an orally bioavailable CXCR4 antagonist. Antiviral Res. 57, A39–A39 12924347

[34] Dar A., Schajnovitz A., Lapid K., Kalinkovich A., Itkin T., Ludin A. et al. (2011) Rapid mobilization of hematopoietic progenitors by AMD3100 and catecholamines is mediated by CXCR4-dependent SDF-1 release from bone marrow stromal cells. Leukemia 25, 1286–1296 10.1038/leu.2011.62 21494253 PMC4175714

[35] DiPersio J.F., Stadtmauer E.A., Nademanee A., Micallef I.N.M., Stiff P.J., Kaufman J.L. et al. (2009) Plerixafor and G-CSF versus placebo and G-CSF to mobilize hematopoietic stem cells for autologous stem cell transplantation in patients with multiple myeloma. Blood 113, 5720–5726 10.1182/blood-2008-08-174946 19363221

[36] DiPersio J.F., Micallef I.N., Stiff P.J., Bolwell B.J., Maziarz R.T., Jacobsen E. et al. (2009) Phase III Prospective Randomized Double-Blind Placebo-Controlled Trial of Plerixafor Plus Granulocyte Colony-Stimulating Factor Compared With Placebo Plus Granulocyte Colony-Stimulating Factor for Autologous Stem-Cell Mobilization and Transplantation for Patients With Non-Hodgkin's Lymphoma. J. Clin. Oncol. 27, 4767–4773 10.1200/JCO.2008.20.7209 19720922

[37] Moyle G., DeJesus E., Boffito M., Wong R.S., Gibney C., Badel K. et al. (2009) Proof of activity with AMD11070, an orally bioavailable inhibitor of CXCR4-tropic HIV type 1. Clin. Infect. Dis. 48, 798–805 10.1086/597097 19193109

[38] O'Hara M.H., Messersmith W., Kindler H., Zhang W., Pitou C., Szpurka A.M. et al. (2020) Safety and Pharmacokinetics of CXCR4 Peptide Antagonist, LY2510924, in Combination with Durvalumab in Advanced Refractory Solid Tumors. J. Pancreat Cancer 6, 21–31 10.1089/pancan.2019.0018 32219196 PMC7097682

[39] Boddu P., Borthakur G., Koneru M., Huang X., Naqvi K., Wierda W. et al. (2018) Initial Report of a Phase I Study of LY2510924, Idarubicin, and Cytarabine in Relapsed/Refractory Acute Myeloid Leukemia. Front. Oncol. 8, 369 10.3389/fonc.2018.00369 30319961 PMC6167965

[40] Salgia R., Stille J.R., Weaver R.W., McCleod M., Hamid O., Polzer J. et al. (2017) A randomized phase II study of LY2510924 and carboplatin/etoposide versus carboplatin/etoposide in extensive-disease small cell lung cancer. Lung Cancer 105, 7–13 10.1016/j.lungcan.2016.12.020 28236984

[41] Bihorel S., Raddad E., Fiedler-Kelly J., Stille J.R., Hing J. and Ludwig E. (2017) Population pharmacokinetic and pharmacodynamic modeling of LY2510924 in patients with advanced cancer. CPT Pharmacometrics Syst. Pharmacol. 6, 614–624 10.1002/psp4.12221 28643374 PMC5613202

[42] Hainsworth J.D., Reeves J.A., Mace J.R., Crane E.J., Hamid O., Stille J.R. et al. (2016) A Randomized, Open-Label Phase 2 Study of the CXCR4 Inhibitor LY2510924 in Combination with Sunitinib Versus Sunitinib Alone in Patients with Metastatic Renal Cell Carcinoma (RCC). Target. Oncol. 11, 643–653 10.1007/s11523-016-0434-9 27154357

[43] Mao Y., Meng Q., Song P., Zhu S., Xu Y., Snyder E.Y. et al. (2018) Novel Bivalent and D-Peptide Ligands of CXCR4 Mobilize Hematopoietic Progenitor Cells to the Blood in C3H/HeJ Mice. Cell Transplant. 27, 1249–1255 10.1177/0963689718784957 29991278 PMC6434473

[44] Huang Y., Huang Z., An J. and Xu Y. (2019) A novel dimeric CXCR4 antagonist synergizes with chemotherapy in acute myeloid leukaemia by mobilizing leukaemic cells from their associated bone marrow niches. Br. J. Haematol. 187, e11–e15 10.1111/bjh.16127 31388999

[45] Smith N., Rodero M.P., Bekaddour N., Bondet V., Ruiz-Blanco Y.B., Harms M. et al. (2019) Control of TLR7-mediated type I IFN signaling in pDCs through CXCR4 engagement-A new target for lupus treatment. Sci. Adv. 5, eaav9019 10.1126/sciadv.aav9019 31309143 PMC6620093

[46] Liu D., Madani N., Li Y., Cao R., Choi W.-T., Kawatkar S.P. et al. (2007) Crystal structure and structural mechanism of a novel anti-human immunodeficiency virus and D-Amino acid-containing chemokine. J. Virol. 81, 11489–11498 10.1128/JVI.02845-06 17686848 PMC2045531

[47] Fernandis A.Z., Cherla R.P. and Ganju R.K. (2003) Differential regulation of CXCR4-mediated T-cell chemotaxis and mitogen-activated protein kinase activation by the membrane tyrosine phosphatase, CD45. J. Biol. Chem. 278, 9536–9543 10.1074/jbc.M211803200 12519755

[48] Princen K., Hatse S., Vermeire K., De Clercq E. and Schols D. (2003) Evaluation of SDF-1/CXCR4-induced Ca2+ signaling by fluorometric imaging plate reader (FLIPR) and flow cytometry. Cytometry A. 51, 35–45 10.1002/cyto.a.10008 12500303

[49] Fricker S.P., Anastassov V., Cox J., Darkes M.C., Grujic O., Idzan S.R. et al. (2006) Characterization of the molecular pharmacology of AMD3100: a specific antagonist of the G-protein coupled chemokine receptor, CXCR4. Biochem. Pharmacol. 72, 588–596 10.1016/j.bcp.2006.05.010 16815309

[50] Wong R.S., Bodart V., Metz M., Labrecque J., Bridger G. and Fricker S.P. (2008) Comparison of the potential multiple binding modes of bicyclam, monocylam, and noncyclam small-molecule CXC chemokine receptor 4 inhibitors. Mol. Pharmacol. 74, 1485–1495 10.1124/mol.108.049775 18768385

[51] Venkatesan S., Rose J.J., Lodge R., Murphy P.M. and Foley J.F. (2003) Distinct mechanisms of agonist-induced endocytosis for human chemokine receptors CCR5 and CXCR4. Mol. Biol. Cell 14, 3305–3324 10.1091/mbc.e02-11-0714 12925765 PMC181569

[52] Hatse S., Princen K., Gerlach L.O., Bridger G., Henson G., De Clercq E. et al. (2001) Mutation of Asp(171) and Asp(262) of the chemokine receptor CXCR4 impairs its coreceptor function for human immunodeficiency virus-1 entry and abrogates the antagonistic activity of AMD3100. Mol. Pharmacol. 60, 164–173 10.1124/mol.60.1.164 11408611

[53] Gerlach L.O., Skerlj R.T., Bridger G.J. and Schwartz T.W. (2001) Molecular interactions of cyclam and bicyclam non-peptide antagonists with the CXCR4 chemokine receptor. J. Biol. Chem. 276, 14153–14160 10.1074/jbc.M010429200 11154697

[54] Rosenkilde M.M., Gerlach L.O., Hatse S., Skerlj R.T., Schols D., Bridger G.J. et al. (2007) Molecular mechanism of action of monocyclam versus bicyclam non-peptide antagonists in the CXCR4 chemokine receptor. J. Biol. Chem. 282, 27354–27365 10.1074/jbc.M704739200 17599916

[55] Carnec X., Quan L., Olson W.C., Hazan U. and Dragic T. (2005) Anti-CXCR4 monoclonal antibodies recognizing overlapping epitopes differ significantly in their ability to inhibit entry of human immunodeficiency virus type 1. J. Virol. 79, 1930–1933 10.1128/JVI.79.3.1930-1933.2005 15650218 PMC544137

[56] Cho B.S., Zeng Z., Mu H., Wang Z., Konoplev S., McQueen T. et al. (2015) Antileukemia activity of the novel peptidic CXCR4 antagonist LY2510924 as monotherapy and in combination with chemotherapy. Blood 126, 222–232 10.1182/blood-2015-02-628677 26031918 PMC4497963

[57] Kufareva I., Rueda M., Katritch V., Stevens R.C., Abagyan R. and participants, G. D. (2011) Status of GPCR modeling and docking as reflected by community-wide GPCR Dock 2010 assessment. Structure 19, 1108–1126 10.1016/j.str.2011.05.012 21827947 PMC3154726

[58] Zhu S., Meng Q., Schooley R.T., An J., Xu Y. and Huang Z. (2019) Structural and biological characterizations of novel high-affinity fluorescent probes with overlapped and distinctive binding regions on CXCR4. Molecules 24, 2928 10.3390/molecules24162928 31412600 PMC6720714

[59] Cox B.D., Prosser A.R., Katzman B.M., Alcaraz A.A., Liotta D.C., Wilson L.J. et al. (2014) Anti-HIV small-molecule binding in the peptide subpocket of the CXCR4:CVX15 crystal structure. Chem. Bio. Chem. 15, 1614–1620 10.1002/cbic.201402056 PMC5776682 24990206

[60] Zhang C., Du C., Feng Z., Zhu J. and Li Y. (2015) Hologram quantitative structure activity relationship, docking, and molecular dynamics studies of inhibitors for CXCR4. Chem. Biol. Drug Des. 85, 119–136 10.1111/cbdd.12377 24923360

[61] Kawatkar S.P., Yan M.C., Gevariya H., Lim M.Y., Eisold S., Zhu X.J. et al. (2011) Computational analysis of the structural mechanism of inhibition of chemokine receptor CXCR4 by small molecule antagonists. Exp. Biol. Med. 236, 844–850 10.1258/ebm.2011.010345 PMC3900290 21697335

[62] CXCR4-AMD11070 complex. PMDB id: PM0084214 http://srv00.recas.ba.infn.it/PMDB/show_complex.php?model=84214

[63] Xu Y.N., Tamamura H., Arakaki R., Nakashima H., Zhang X.Y., Fujii N. et al. (1999) Marked increase in anti-HIV activity, as well as inhibitory activity against HIV entry mediated by CXCR4, linked to enhancement of the binding ability of tachyplesin analogs to CXCR4. AIDS Res. Hum. Retroviruses 15, 419–427 10.1089/088922299311169 10195751

[64] Tamamura H., Hiramatsu K., Mizumoto M., Ueda S., Kusano S., Terakubo S. et al. (2003) Enhancement of the T140-based pharmacophores leads to the development of more potent and bio-stable CXCR4 antagonists. Org. Biomol. Chem. 1, 3663–3669 10.1039/b306613b 14649897

[65] Zachariassen Z.G., Thiele S., Berg E.A., Rasmussen P., Fossen T., Rosenkilde M.M. et al. (2014) Design, synthesis, and biological evaluation of scaffold-based tripeptidomimetic antagonists for CXC chemokine receptor 4 (CXCR4). Bioorg. Med. Chem. 22, 4759–4769 10.1016/j.bmc.2014.07.004 25082513

[66] Doranz B.J., Grovit-Ferbas K., Sharron M.P., Mao S.H., Goetz M.B., Daar E.S. et al. (1997) A small-molecule inhibitor directed against the chemokine receptor CXCR4 prevents its use as an HIV-1 coreceptor. J. Exp. Med. 186, 1395–1400 10.1084/jem.186.8.1395 9334380 PMC2199097

[67] Tahirovic Y.A., Pelly S., Jecs E., Miller E.J., Sharma S.K., Liotta D.C. et al. (2020) Small molecule and peptide-based CXCR4 modulators as therapeutic agents. A patent review for the period from 2010 to 2018. Expert Opin. Ther. Pat. 1–15 10.1080/13543776.2020.1707186 31854208

[68] Rosenberg E.M. Jr, Harrison R.E.S., Tsou L.K., Drucker N., Humphries B., Rajasekaran D. et al. (2019) Characterization, dynamics, and mechanism of CXCR4 antagonists on a constitutively active mutant. Cell Chem. Biol. 26, 662–673 10.1016/j.chembiol.2019.01.012 30827936 PMC6736600

[69] Jorgensen A.S., Daugvilaite V., De Filippo K., Berg C., Mavri M., Benned-Jensen T. et al. (2021) Biased action of the CXCR4-targeting drug plerixafor is essential for its superior hematopoietic stem cell mobilization. Commun. Biol. 4, 569 10.1038/s42003-021-02070-9 33980979 PMC8115334

[70] Thiele S., Mungalpara J., Steen A., Rosenkilde M.M. and Vabeno J. (2014) Determination of the binding mode for the cyclopentapeptide CXCR4 antagonist FC131 using a dual approach of ligand modifications and receptor mutagenesis. Br. J. Pharmacol. 171, 5313–5329 10.1111/bph.12842 25039237 PMC4294042

[71] Tian S., Choi W.T., Liu D., Pesavento J., Wang Y., An J. et al. (2005) Distinct functional sites for human immunodeficiency virus type 1 and stromal cell-derived factor 1alpha on CXCR4 transmembrane helical domains. J. Virol. 79, 12667–12673 10.1128/JVI.79.20.12667-12673.2005 16188969 PMC1235829

[72] Zhou N., Luo Z., Luo J., Liu D., Hall J.W., Pomerantz R.J. et al. (2001) Structural and functional characterization of human CXCR4 as a chemokine receptor and HIV-1 co-receptor by mutagenesis and molecular modeling studies. J. Biol. Chem. 276, 42826–42833 10.1074/jbc.M106582200 11551942

[73] Fang X., Meng Q., Zhang H., Liang B., Zhu S., Wang J. et al. (2020) Design, synthesis, and biological characterization of a new class of symmetrical polyamine-based small molecule CXCR4 antagonists. Eur. J. Med. Chem. 200, 112410 10.1016/j.ejmech.2020.112410 32492596

[74] Das R. and Baker D. (2008) Macromolecular modeling with rosetta. Annu. Rev. Biochem. 77, 363–382 10.1146/annurev.biochem.77.062906.171838 18410248

[75] Tyka M.D., Keedy D.A., Andre I., Dimaio F., Song Y., Richardson D.C. et al. (2011) Alternate states of proteins revealed by detailed energy landscape mapping. J. Mol. Biol. 405, 607–618 10.1016/j.jmb.2010.11.008 21073878 PMC3046547

[76] Conway P., Tyka M.D., DiMaio F., Konerding D.E. and Baker D. (2014) Relaxation of backbone bond geometry improves protein energy landscape modeling. Protein Sci. 23, 47–55 10.1002/pro.2389 24265211 PMC3892298

[77] Raveh B., London N. and Schueler-Furman O. (2010) Sub-angstrom modeling of complexes between flexible peptides and globular proteins. Proteins 78, 2029–2040 10.1002/prot.22716 20455260

[78] Raveh B., London N., Zimmerman L. and Schueler-Furman O. (2011) Rosetta FlexPepDock ab-initio: simultaneous folding, docking and refinement of peptides onto their receptors. PloS ONE 6, e18934 10.1371/journal.pone.0018934 21572516 PMC3084719

