# Supplementary material for: Biological and mutational analyses of CXCR4–antagonist interactions and design of new antagonistic analogs
Source: Biosci Rep. 2023 Dec 22;43(12):BSR20230981. doi: 10.1042/BSR20230981 (PMC10987480; doi:10.1042/BSR20230981)
Supplement: Supplementary Figure S1-S2 and Tables S1-S3 [file BSR-2023-0981_supp.pdf]

Table S1 Primer for CXCR4-EGFP conjugate

Primer	Sequence (5'to3')
CXCR4 forward primer	GCGAATTCATGTCCATTCCTTTGCCTCTTTTGC
CXCR4 reverse primer	GCTCTAGAGCTGGAGTGAAAACTTGAAGAC
EGFP forward primer	GGTCTAGAGTGAGCAAGGGCGAGGAGCTGTTCAC
EGFP reverse primer	CTAATGGGCCCCTTGTACAGCTCGTCCATGCCGAGAGTGATCCCG

Table S2 Primer for mutated CXCR4

Primer	Sequence (5'to3')
R30A-forward	GTTTCGCTGAAGAAAATGCTAATTTCAATAAAATCTTCC
R30A-reverse	CATTTTCTTCAGCGAAACAGGGTTCCTTCATGGAGTCATAG
Y45A-forward	CACCATCGCCTCCATCATCTTCTTAAC
Y45A-reverse	GATGGAGGCGATGGTGGGCAGGAAGAT
F87A-forward	CCTCCTCGCTGTCATCACGCTTCCCTTCTGGGCAGTTG
F87A-reverse	GTGATGACAGCGAGGAGGTCGGCCACTGACAGGTGCAGC
W94A-forward	CCTTCGCGGCAGTTGATGCCGTGGCAAACTGGTACTTTG
W94A-reverse	CATCAACTGCCGCGAAGGGAAGCGTGATGACAAAGAGG
D97A-forward	GCAGTTGCCGCCGTGGCAAACTGGTACTTTG
D97A-reverse	CACGGCGGCAACTGCCCAGAAGGGAAGCGTG
V112A-forward	CAAGGCAGCCCATGTCATCTACACAGT
V112A-reverse	GACATGGGCTGCCTTGCATAGGAAGTTC
H113A-forward	GGCAGTCGCTGTCATCTACACAGTCAACCTCTACAG
H113A-reverse	GATGACAGCGACTGCCTTGCATAGGAAGTTCCCAAAG
Y116A-forward	GTCATCGCCACAGTCAACCTCTACAGCAG
Y116A-reverse	GACTGTGGCGATGACATGGACTGCCTTG
T117A-forward	GTCATCTACGCAGTCAACCTCTACAGCAG
T117A-reverse	GACTGCGGCGATGACATGGACTGCCTTG
N119A-forward	CACAGTCGCCCTCTACAGCAGTGTCCT
N119A-reverse	GTAGAGGGCGACTGTGTAGATGACATG
W161A-forward	GCGTCGCGATCCCTGCCCTCCTGCT
W161A-reverse	CAGGGATCGCGACGCCAACATAGACC
D171A-forward	CTATTCCCGCCTTCATCTTTGCCAACGTCAGTGAGGCAG
D171A-reverse	GATGAAGGCGGGAATAGTCAGCAGGAGGGCAGGGATCCAG
D193A-forward	CCCAATGCCTTGTGGGTGGTTGTGTTCCAGTTTCAGC
D193A-reverse	CCACAAGGCATTGGGGTAGAAGCGGTCACAGATATATC
W195A-forward	GACTTGGCTGTGGTTGTGTTCCAGTTTCAGCACATCATGG
W195A-reverse	CAACCACAGCCAAGTCATTGGGGTAGAAGCGGTCACAG

Table S3 Primer for mutated CXCR4

Primer	Sequence (5'to3')
Q200A-forward	GTGTTCGCGTTTCAGCACATCATGGT
Q200A-reverse	GCTGAAACGCGAACACAACCACCCAC
H203A-forward	GTTTCAGGCCATCATGGTTGGCCTTATCCT
H203A-reverse	CCATGATGGCCTGAAACTGGAACACAACCA
W252A-forward	CCTGTGCCCTGCCTTACTACATTGGGAT
W252A-reverse	GTAAGGCAGGGCACAGGCGAAGAAAGCC
Y255A-forward	CTGCCTTACGCCATTGGGATCAGCATCGACT
Y255A-reverse	CAATGGCGTAAGGCAGCCAACAGGCGAAG
Y256A-forward	CTGCCTGCCTACATTGGGATCAGCATCGACT
Y256A-reverse	CAATGTAGGCAGGCAGCCAACAGGCGAAG
I259A-forward	CATTGGGGCCAGCATCGACTCCTTCAT
I259A-reverse	GATGCTGGCCCCAATGTAGTAAGGCAG
D262N-forward	GCATCAACTCCTTCATCCTCCTGGAAATCATCAAGCAAG
D262N-reverse	GATGAAGGAGTTGATGCTGATCCCAATGTAGTAAG
D262E-forward	GCATCGAGTCCTTCATCCTCCTGGAAATCATCAAGCAAG
D262E-reverse	GATGAAGGACTCGATGCTGATCCCAATGTAGTAAG
H281A-forward	CACTGTGGCCAAGTGGATTTCCATCAC
H281A-reverse	CACTTGGCCACAGTGTTCTCAAACT
I284A-forward	CAAGTGGGCTTCCATCACCGAGGCCCT
I284A-reverse	GATGGAAGCCCACTTGTGCACAGTGTT
E288D-forward	CATCACCAACGCCCTAGCTTTCTTCCACTGTT
E288D-reverse	CTAGGGCGTTGGTGATGGAAATCCACTTGTGCA
E288Q-forward	CATCACCCAGGCCCTAGCTTTCTTCCACTGTTGTC
E288Q-reverse	CTAGGGCCTGGGTGATGGAAATCCACTTGTGCA
E288A-forward	CATCACCGCCGCCCTAGCTTTCTTCCACTGTTGTC
E288A-reverse	CTAGGGCGGCGGTGATGGAAATCCACTTGTGCA

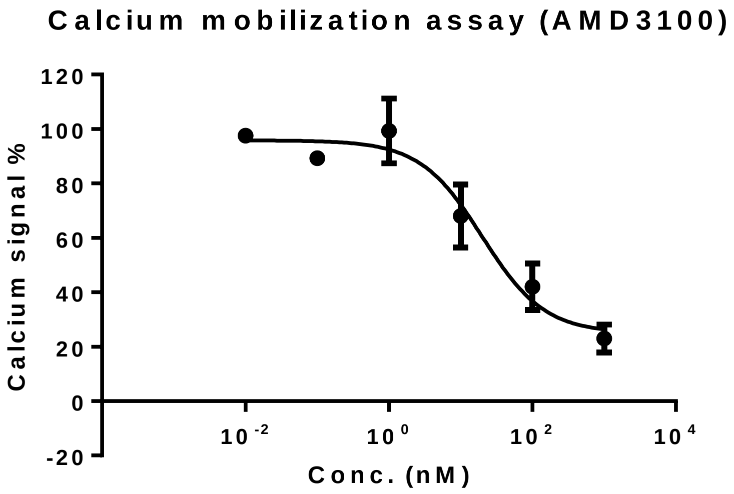

Fig. S1. The inhibition of AMD3100 on SDF-1α-CXCR4 induced intracellular calcium mobilization.

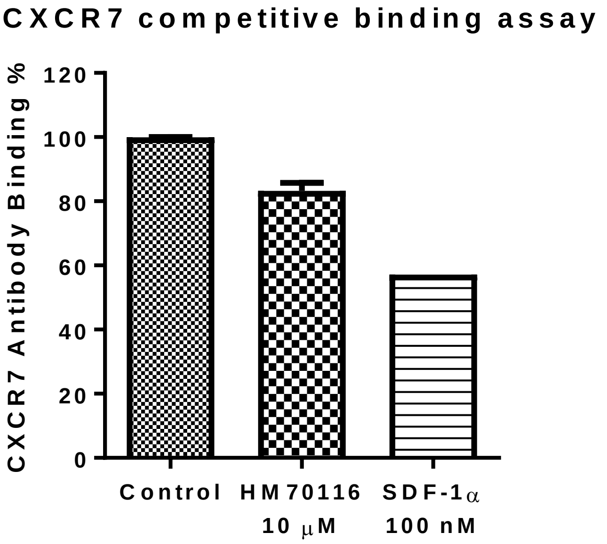

Fig. S2. The competitive binding affinity of HM70116 to CXCR7. HM70116 (10μM) was incubated with HEK293 cells expressing CXCR7 and its antibody 11G8 (2.5μg/ml) to detect the competitive binding activity of HM70116 to the receptor. 11G8 was a specific antibody of CXCR7 and binds to N-terminus residue D16 and ECL1 residue E114 of CXCR7 that different from the binding site of its natural ligand CXCL12 interacting with residues D179 and D275 of CXCR7[1]. Among them, the group without HM70116 and only 11G8 was 100%, and the group without HM70116 and 12G5 was 0%. FITC-labeled secondary antibody IgG molecules were added to all groups to exclude the interference of non-specific binding. The experiment was average of three independent experiments.

1 Benredjem, B., Girard, M., Rhainds, D., St-Onge, G. and Heveker, N. (2017) Mutational Analysis of Atypical Chemokine Receptor 3 (ACKR3/CXCR7) Interaction with Its Chemokine Ligands CXCL11 and CXCL12. J Biol Chem. **292**, 31-42
